# Harmonic balance analysis of magnetically coupled two-degree-of-freedom bistable energy harvesters

**DOI:** 10.1038/s41598-022-10061-x

**Published:** 2022-04-13

**Authors:** Jinhong Noh, Minh Sang Nguyen, Pilkee Kim, Yong-Jin Yoon

**Affiliations:** 1 Department of Mechanical Engineering, Korea Advanced Institute of Science and Technology, 291 Daehak-ro, Daejeon, 34141 South Korea; 2 School of Mechanical Design Engineering, College of Engineering, Jeonbuk National University, 567 Baekje-daero, Deokjin-gu, Jeonju-si, 54896 South Korea; 3 Singapore Institute of Manufacturing Technology (SIMTech), 71 Nanyang Dr., Singapore, 638075 Singapore; 4 Eco-Friendly Machine Parts Design Research Center, Jeonbuk National University, 567 Baekje-daero, Deokjin-gu, Jeonju-si, 54896 South Korea

**Keywords:** Mechanical engineering, Energy harvesting

## Abstract

Because a magnetically coupled two-degree-of-freedom bistable energy harvester (2-DOF MCBEH) shows the rich, complicated nonlinear behaviors caused by its coupled cubic nonlinearities, understanding the dynamics remains challenging. This paper reports and investigates the important nonlinear dynamical phenomena of the 2-DOF MCBEHs by performing the harmonic balance analysis (HBA). All periodic solution branches are identified in order to study and comprehend the complicated dynamics of the 2-DOF MCBEHs. This end requires care when truncating the harmonic balance solution. For a 1-DOF MCBEH, which is the conventional type, the fundamental harmonic is able to approximately describe the steady-state periodic response. However, high-order harmonics are significant for the 2-DOF MCBEH. This paper demonstrates that the harmonic balance solution should involve the high-order terms instead of using the oversimplified single-harmonic solution. By performing the proposed HBA, important solution branches are reported, and their dynamical behaviors are studied. Moreover, the complete architecture of the frequency response of the 2-DOF MCBEH is disclosed across the entire frequency range. The HBA also reveals the underlying physics of building a bridge between the first and second primary resonant areas under a strong excitation. In the future, the findings in the present report can be utilized in the design process of the 2-DOF MCBEHs.

## Introduction

Energy harvesting technology has been noteworthy as one of the alternative ways to power electronic devices instead of batteries. For example, in the Internet of Things networks, the maintenance cost of battery replacement is reduced by scavenging ambient energy with energy harvesting^1^. In addition, energy harvesters are sustainable and eco-friendly, whereas batteries require recyclability due to its sustainability concerns^2,3^. Furthermore, energy harvesting is the more promising alternative while replacing or recharging batteries are demanding, impractical, or hazardous^4^.

Among the energy sources from the ambient environment, mechanical vibration has been widely utilized for its accessibility^5^. Piezoelectric material has been commonly utilized to convert strain energy caused by mechanical vibration into electrical voltage for power supply^6^. In the early stages of the development in piezoelectric vibratory energy harvesters, the design principle was based on the linear resonance^7^. However, the main drawback of the linear harvesters is that the steady-state performance bandwidth is too narrow because the excitation frequency is inevitably detuned in real applications^8^. To achieve broadband performance, energy harvesters, of which frequency responses have softening or hardening characteristics, have been developed by introducing nonlinearities into the system stiffness^9,10^.

In this regard, many researchers have been trying to develop bistable energy harvesters (BEHs) of which restoring force potential is double-well shape^11,12^. When the BEH vibrates within one well, the oscillating behavior is called intrawell oscillation. On the other hand, interwell oscillation indicates the large orbit oscillation in which the state overcomes the saddle barrier between the wells and oscillates across the wells. Because the higher energy output is obtained when the interwell motion occurs compared to the intrawell motion, the design endeavor to achieve the interwell dynamics has been focused^13,14^.

In this paper, two contrasting architectures of BEHs are considered: a conventional BEH and a magnetically coupled two-degree-of-freedom bistable energy harvester (2-DOF MCBEH). As introduced in the following section, these BEHs consist of the permanent magnets for stiffness nonlinearities and the clamped rectangular bimorph beams for transducing, typically used in piezoelectric energy harvesting^15^. The main difference between the two configurations is that the 2-DOF MCBEH has another oscillating bimorph with the tip magnet, whereas in the conventional BEH, this magnet is fixed to the base structure. Due to this additional degree of freedom, the second primary intrawell resonance exists, in which the in-phase mode regime dominates. This resonance offers another route to the high-energy orbit motion because the state is likely to surmount the saddle barrier around the intrawell resonance^16^. Moreover, because the two primary resonances are possibly connected through other interwell motions in frequency response space, the broadband performance can be further enhanced for the 2-DOF MCBEH^17^.

Harmonic balance analysis (HBA) has been widely performed to investigate dynamical behaviors of cantilever-type piezoelectric energy harvesters of which stiffness nonlinearities are imposed by permanent magnets^18–28^. For example, for the type of the conventional BEHs studied in this paper, Stanton et al. demonstrated that the HBA is accurate when compared to the numerically obtained results and stated that the HBA can be utilized in the design optimization process for its fast computation^29^. In addition, Barton et al. applied the HBA to verify their sophisticated experimental method which reveals the bifurcation structure by finding both the stable and the unstable solution branches, and the results showed qualitative agreement^30^. However, because all of these HBA studies approximated the solution form up to the first-order expansion, describing superharmonic behavior was not allowed. To overcome this limitation, Harne and Wang implemented the HBA with fundamental and superharmonic components to investigate the characteristics of the superharmonic behavior^31^. It shows that for the HBA, using a suitable solution form is important according to what dynamical behavior is investigated.

For the 2-DOF MCBEHs, Lan et al. assumed the single-harmonic solution in the HBA, neglecting the effects of high-order harmonics^32^. With this first-order truncation, they concluded that the voltage output of one beam in interwell motion at the first primary resonance decreases as excitation amplitude increases. This conclusion was named *trade-off* by these authors. However, this statement was based on the HBA with the single-harmonic solution. In fact, for the 2-DOF case, an assumed solution of the first primary resonant behavior should involve the high-order harmonics up to at least the third-order because third-harmonic distortions produce significant effects^33^. Moreover, Lan et al. reported that three and two solution branches exist in the first and second resonances, respectively. However, when the HBA is conducted, other solution branches should be found, as shown later in the present report. Furthermore, the oversimplified solution form cannot reveal multiple-period solution branches which should be studied especially in the 2-DOF case to examine their bridging performance^17^.

The present study resolves the above-mentioned limitations and reveals all solution branches which are necessary to understand rich, complicated dynamics of the 2-DOF MCBEHs by performing HBA. An ansatz is established to involve high-order harmonics and multiple-period motions. Firstly, it is demonstrated that the first-order truncation is inappropriate to represent the first primary resonant behavior. Subsequently, this paper provides two examples to demonstrate that the previously reported *trade-off* concept is incorrect or negligible. Secondly, a symmetrical interwell solution branch is reported and its dynamical behavior is investigated because this branch is promising for broadband energy harvesting. Thirdly, the second primary resonance is studied, and the HBA study reveals all coexisting branch structures. Lastly, it is identified that the multiple-period oscillations are not suitable to be utilized for a bridge between the first and second primary resonant ranges for wideband harvesting performance. Instead, the long interwell solution branch, newly investigated in the present paper, is promising, and the bridging behavior of this branch is reported.

## Mathematical model

**Figure 1:**
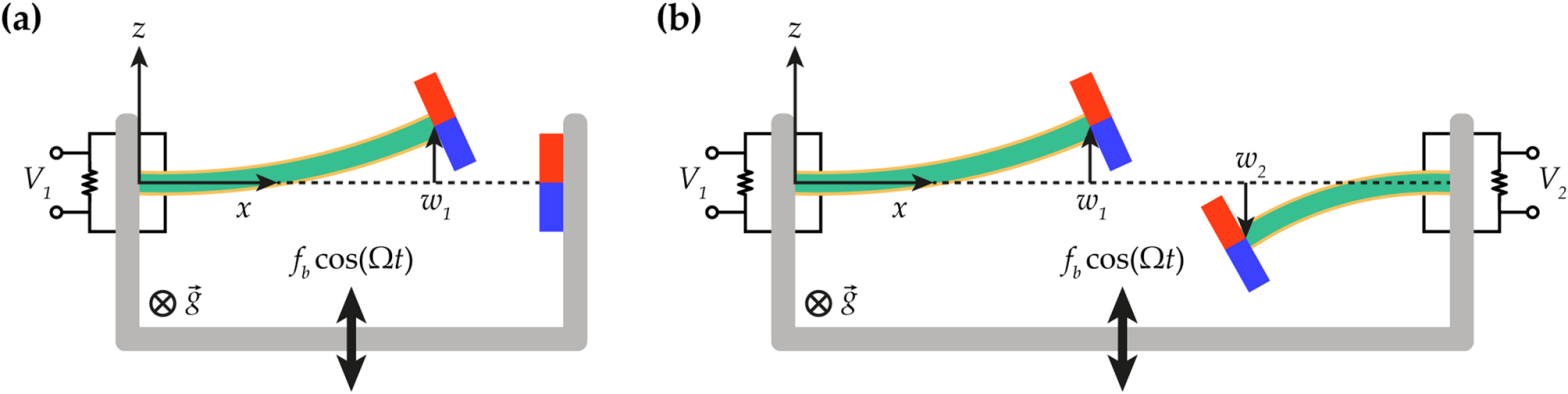
**(a)** Schematic of a conventional bistable energy harvester (BEH). **(b)** Schematic of two-degree-of-freedom magnetically coupled bistable energy harvester (2-DOF MCBEH). In **(a)**, one bimorph beam with a tip magnet oscillates, and another magnet is attached to the rigid base structure. In **(b)**, two bimorph beams with tip magnets oscillate. As a representative, the out-of-phase motion is illustrated. The red and blue areas of the magnets indicate the N-pole and the S-pole, respectively. The harmonic base excitation is formulated by $f_{b} \cos (\Omega t)$. $w_{1}$ and $w_{2}$ are the deflections of the beams at the tips with respect to the neutral surface. Voltage outputs across the load resistances are denoted by $V_{1}$ and $V_{2}$. Gravitational force acts in the direction of the *y*-axis.

Figure 1 shows the schematic diagrams of the conventional BEH and 2-DOF MCBEH. The conventional harvester has one cantilever beam with a tip magnet and another magnet fixed on the rigid frame at the same height, whereas the 2-DOF MCBEH has two cantilever beams with tip magnets. The separation distance defines the distance between the centers of the two magnets which is measured when the beams are undeformed. This distance is set to induce the magnetic coupling force to impose the static bistability. The longer beam on the left in Fig. 1b and the shorter beam on the right are called Beam 1 and Beam 2, respectively. The beams consist of a metal substrate and two piezoelectric layers bonded on the top and bottom surfaces of the substrate. Load resistances are connected to the piezoelectric layers in series. The coordinate system is located at the center of the cross-section and clamped position of Beam 1 as illustrated in Fig. 1. Because gravity points to the *y*-direction, it is neglected in the mathematical modeling process. Under harmonic base excitation on the rigid frame, the tips of the beams oscillate in the *z*-direction, and deflections of Beam 1 and Beam 2 are denoted by $w_{1}$ and $w_{2}$, respectively. At the same time, owing to the piezoelectricity, voltage outputs, $V_{1}$ and $V_{2}$ for Beam 1 and Beam 2 respectively, are generated across the load resistances. The base excitation is formulated as $f_{b} \cos (\Omega t)$ where $f_{b}$, $\Omega$, and $t$ are the acceleration, excitation frequency, and time, respectively. For numerical and theoretical simulations, it is assumed that the base of the energy harvester is excited by a harmonic force or a swept-sine force, which possibly occurs in practical applications such as a rotary pump, a car engine, or a bridge^34,35^. The mathematical modeling process for the beams is conducted by applying the linear elastic, linear piezoelectricity, and Euler–Bernoulli beam theories. Kirchhoff’s law is used for the circuits and the magnetic charge model is utilized for the magnetic coupling force. With consideration of the inertia of the tip magnet, the governing field equation is derived by employing Hamilton’s principle, and after carrying out the modal analysis and the discretization process, it is reduced to an oscillator model such that

1 $$\ddot{w}_{i} + 2\zeta_{i} \omega_{i} \dot{w}_{i} + \omega_{i}^{2} w_{i} - \beta_{i} V_{i} - f_{mi} = - \alpha_{i} f_{b} \cos (\Omega t)\;\;\;{\kern 1pt} \left( {i = 1,2} \right),$$

2 $$\dot{V}_{i} + \eta_{i} V_{i} + \gamma_{i} \dot{w}_{i} = 0\;\;\;{\kern 1pt} \left( {i = 1,2} \right),$$

where $\zeta_{i}$ is the damping ratio, $\omega_{i}$ is the natural frequency, $\beta_{i}$ and $\gamma_{i}$ are the electromechanical coupling coefficients, $\alpha_{i}$ is the coefficient for mass normalization, $\eta_{i}$ is the modal resistance, and a dot indicates a time derivative. Herein, the magnetic coupling force, denoted by $f_{mi}$, brings position-dependent nonlinearities. The magnetic coupling force was truncated by the Taylor series expansion up to the third order with respect to zero as follow:

3 $$\begin{aligned} f_{mi} (w_{1} ,w_{2} ) = & \left. {\frac{{\partial F_{mi} }}{{\partial w_{1} }}} \right|_{(0,0)} w_{1} + \left. {\frac{{\partial F_{mi} }}{{\partial w_{2} }}} \right|_{(0,0)} w_{2} \\ & + \frac{1}{6}\left. {\frac{{\partial^{3} F_{mi} }}{{\partial w_{1}^{3} }}} \right|_{(0,0)} w_{1}^{3} + \frac{1}{2}\left. {\frac{{\partial^{3} F_{mi} }}{{\partial w_{1}^{2} \partial w_{2} }}} \right|_{(0,0)} w_{1}^{2} w_{2} + \frac{1}{2}\left. {\frac{{\partial^{3} F_{mi} }}{{\partial w_{1} \partial w_{2}^{2} }}} \right|_{(0,0)} w_{1} w_{2}^{2} + \frac{1}{6}\left. {\frac{{\partial^{3} F_{mi} }}{{\partial w_{2}^{3} }}} \right|_{(0,0)} w_{2}^{3} , \\ \end{aligned}$$

where $F_{mi}$ is the position-dependent modal magnetic coupling force from the magnetic charge model. Because this truncation yields the cubic nonlinearities, the oscillator model is the 2-DOF Duffing-type oscillator.

The cross-sectional dimensions of the metal substrate and the piezoelectric layers are 10 mm × 0.3 mm and 10 mm × 0.052 mm, respectively. For the metal substrate, density and Young’s modulus are set to be 7850 kg/m^3^ and 200 GPa. For the piezoelectric layer, 1780 kg/m^3^ of density and 3 GPa of Young’s modulus are used. Piezoelectric constant and permittivity are − 23 pm/V and 110 pF/m, respectively. The magnets are made of neodymium with 900 kA/m of magnetization. The dimensions of the magnets are 10 mm × 6 mm × 2 mm (width × thickness × length). The damping ratios are chosen as 0.015. The length of Beam 1 is 70 mm, and the length of Beam 2 is varied as summarized in Table 1. Note that the parameter values for the dimensions and properties of the system are chosen in physically realistic ranges, referring to the previous report where our oscillator model was validated experimentally^17^.

**Table 1:** Simulation conditions used in simulations. Case A in the first row is the case of the conventional BEH. The separation distance is 600-μm ahead of the pitchfork bifurcation point of static equilibrium.

Case	Length (mm)		Length ratio	Natural frequency (rad/s)		Separation distance (mm)
	Beam 1	Beam 2		Beam 1	Beam 2	
A	70	0	–	165.6	–	10.930
B	70	30	0.429	165.6	628.7	11.187
C	70	49	0.700	165.6	293.6	11.819
D	70	64	0.914	165.6	191.5	12.613
E	70	68	0.971	165.6	173.6	12.865
F	70	69	0.986	165.6	169.5	12.924
G	70	70	1	165.6	165.6	12.989

## Simulation methods

### Numerical analysis

Numerical analysis is performed to verify the results of harmonic balance analysis. For direct numerical integration, a state vector is introduced such that

4 $${\mathbf{x}} = \left[ {\begin{array}{*{20}c} {w_{1} } & {w_{2} } & {V_{1} } & {V_{2} } & {\dot{w}_{1} } & {\dot{w}_{2} } \\ \end{array} } \right]^{T} ,$$

where superscript *T* means the transpose. Subsequently, a system of differential equations is given by

5 $${\dot{\mathbf{x}}} = {\mathbf{f}}({\mathbf{x}},t) = \left[\begin{array}{*{20}l} x_{5} \\ x_{6} \\ - \eta_{1} x_{3} - \gamma_{1} x_{5} \\ - \eta_{2} x_{4} - \gamma_{2} x_{6} \\ - \alpha_{1} f_{b} \cos \left( {\Omega t} \right) - 2\zeta_{1} \omega_{1} x_{5} - \omega_{1}^{2} x_{1} + \beta_{1} x_{3} + f_{m1} \\ - \alpha_{2} f_{b} \cos \left( {\Omega t} \right) - 2\zeta_{2} \omega_{2} x_{6} - \omega_{2}^{2} x_{2} + \beta_{2} x_{4} + f_{m2} \\ \end{array} \right].$$

The Runge–Kutta method is applied to Eq. (5) for computation. Sine-swept responses are obtained by numerically integrating the state vector with a linear sweep rate. To obtain a frequency-domain response, the fast Fourier transform (FFT) algorithm is employed.

### Harmonic balance analysis

**Figure 2:**
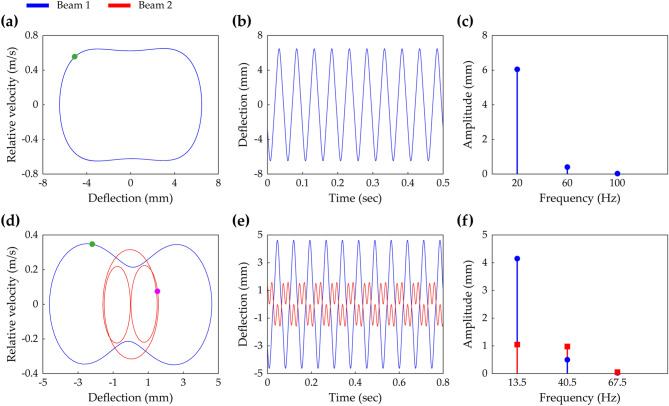
**(a–c)** The steady-state interwell motion of the conventional BEH when the base acceleration is 4 m/s^2^ and the excitation frequency is 20 Hz. **(d–f)** The steady-state interwell motion of the 2-DOF MCBEH when the base acceleration is 7 m/s^2^ and the excitation frequency is 13.5 Hz. The values of Case A and Case C (Table 1) are used to **(a–f)**, respectively. **(a,d)** Show the phase portraits and the stroboscopic points. The time-domain responses are plotted in **(b,e)**, and their corresponding fast Fourier transform (FFT) results are depicted in **(c,f)**.

**Figure 3:**
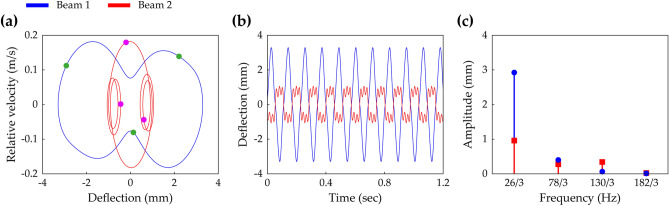
The steady-state interwell motion of the 2-DOF MCBEH with the same conditions of Fig. 2d–f, but herein, the excitation frequency is 26 Hz. **(a)** Shows the phase portrait and the stroboscopic points. **(b,c)** Are the time-domain response and the corresponding FFT result, respectively.

The HBA is performed to reveal all solution branches in the frequency response of the oscillator model described in Eqs. (1) and (2). Achieving this end requires an ansatz, expressed by a Fourier series, and reasonable truncation order for this assumed solution form. Before introducing the ansatz employed in this paper, numerical analysis results are remarked in Figs. 2 and 3.

Figure 2 demonstrates that for the 2-DOF MCBEH, the third-harmonic component of the solution should not be neglected, whereas for the conventional BEH, approximation with only the fundamental term is likely valid. Figure 2a–c show the phase portrait, time-domain response, and corresponding FFT result of the steady-state interwell motion of the conventional BEH, respectively. It implies that for the conventional BEH, the single-harmonic solution is sensible because the time-domain response has the shape of sine function approximately and the third-harmonic component is negligible compared to the fundamental component. However, as shown in Fig. 2d–f, Beam 2 of the 2-DOF MCBEH has the noticeable distorted feature in the time-domain response and the significant magnitude of the third-harmonic component whereas the fundamental harmonic is dominant for Beam 1. This phenomenon has been reported as the third-harmonic distortion in forced oscillation^33^.

Figure 3 suggests that considering up to at least the fifth-order harmonic is recommended to express the period—3* T* oscillation. Here, *T* is defined by 2*π*/Ω. The phase portrait and stroboscopic points of the steady-state interwell period—3* T* motion are depicted in Fig. 3a. The time-domain response and corresponding FFT result are shown in Fig. 3b,c, respectively. The fundamental frequency equals Ω/3 for period—3* T* motion as shown in Fig. 3c. For Beam 1, Fig. 3b,c show that the fundamental harmonic is dominant. However, the response of Beam 2 has both of the third- and fifth-order harmonics of significant magnitude relative to the magnitude of the fundamental harmonic. It is noted that the amplitude of the fifth-order term is larger than the amplitude of the third-order term.

Considering the above-mentioned issues, the steady-state solution is approximately assumed by the following Fourier series:

6 $$w_{i} (t) \approx a_{i0} + \mathop \sum \limits_{k = 1}^{H} \, a_{ick} \cos \left( {\frac{k\Omega t}{n}} \right) + a_{isk} \sin \left( {\frac{k\Omega t}{n}} \right),$$

7 $$V_{i} (t) \approx b_{i0} + \mathop \sum \limits_{k = 1}^{H} \, b_{ick} \cos \left( {\frac{k\Omega t}{n}} \right) + b_{isk} \sin \left( {\frac{k\Omega t}{n}} \right),$$

where *H* is the truncation order and *n* indicates a multiple of the period *T* of the base excitation. The truncation order, *H*, is set to be 5 to involve harmonics up to the fifth order. *n* equals 1, 2, and 3 for period—1* T*, 2* T*, and 3* T* solutions, respectively. Substituting the ansatz, Eqs. (6) and (7), into the oscillator model, Eqs. (1) and (2), leads to balance equations. Because the magnetic coupling force, Eq. (3), was formulated with the cubic nonlinearities, the balance equations are given explicitly by applying the trigonometric identities. The balance equations, which are nonlinear algebraic equations, are solved by implementing the Newton–Raphson method to find the Fourier coefficients in Eqs. (6) and (7) (see Supplementary Information for details). Using Eqs. (6) and (7) enables the HBA to describe the harmonic distortion phenomena and the multiple periodic motions which should be investigated importantly for 2-DOF MCBEHs^17,33^. If an ansatz is truncated up to the first order (*H* = 1) and describes the only period—1* T* motion, it is impossible to describe the complicated dynamics of 2-DOF MCBEHs.

Stability characteristics of a periodic solution obtained by the HBA are investigated according to the Floquet theory. For perturbed dynamics of the periodic orbit, the Jacobian matrix is formulated explicitly as

8 $$\begin{aligned} {\mathbf{J}} &= \frac{{\partial {\mathbf{f}}}}{{\partial {\mathbf{x}}}}{\dot{\mathbf{x}}} \\ &= \left[ {\begin{array}{*{20}l} 0 & 0 & 0 & 0 & 1 & 0 \\ 0 & 0 & 0 & 0 & 0 & 1 \\ 0 & 0 & { - \eta_{1} } & 0 & { - \gamma_{1} } & 0 \\ 0 & 0 & 0 & { - \eta_{2} } & 0 & { - \gamma_{2} } \\ { - \omega_{1}^{2} + \frac{{\partial f_{m1} }}{{\partial w_{1} }}} & {\frac{{\partial f_{m1} }}{{\partial w_{2} }}} & {\beta_{1} } & 0 & { - 2\zeta_{1} \omega_{1} } & 0 \\ {\frac{{\partial f_{m2} }}{{\partial w_{1} }}} & { - \omega_{2}^{2} + \frac{{\partial f_{m2} }}{{\partial w_{2} }}} & 0 & {\beta_{2} } & 0 & { - 2\zeta_{2} \omega_{2} } \\ \end{array} } \right]. \\ \end{aligned}$$

Because the Fourier coefficients are obtained as a result of the HBA, the stability studies are conducted in the frequency domain^36,37^. To determine Floquet exponents, the eigenvector sorting algorithm is applied^38^. The Floquet multipliers, eigenvalues of the monodromy matrix, are calculated with the relation, $\mu = \exp (\nu T)$ where $\mu$ and $\nu$ are the Floquet multiplier and exponent, respectively. The type of local bifurcations in frequency response is confirmed by studying how the Floquet multiplier of the largest magnitude leaves the unit circle.

## Results

Table 2 categorizes every solution branch in frequency response found by performing the proposed HBA. Eight solution branches, from Branch A to H, are period—1* T* oscillations, and the others are multiple periodic oscillations. Because Branch A, B, C, and J oscillate along low energy orbit, confined to one potential well with small amplitude, these intrawell branches are of no particular importance for energy harvesting. Branch D and Branch E are interwell behaviors dominated by the out-of-phase mode dynamics and the in-phase mode dynamics, respectively^16^. In this paper, the regions in which Branch D and Branch E emerge are called the first primary resonance and the second primary resonance, respectively. In these two resonances, interwell motions asymmetrical with respect to **x** = **0** are reported. Branch G is the period—1* T* asymmetrical interwell solution branch which appears in the first primary resonance, and Branch I is the period—2* T* asymmetrical interwell solution branch in the second primary resonance. More importantly, this paper reports Branch F, a long, large symmetrical interwell solution branch, isolated between or across the first and second primary resonances. Because this branch exists across a wide frequency range with large amplitude, the dynamical behavior should be revealed in terms of the broadband performance of energy harvesting. Disconnected frequency bandwidth exists when instabilities occur in the middle of Branch F as shown later, and Branch H, asymmetrical interwell motion, is found within this disconnected bandwidth. Lastly, Branch K and Branch L, period—3* T* interwell oscillations, are reported. Whereas Branch K is symmetrical, Branch L is asymmetrical with respect to **x** = **0**.

**Table 2:** Solution branches obtained by the harmonic balance analysis (HBA).

Branch	Period	Intrawell	Interwell	Sym.	Asym.	Location	Color in sweep response (rgb)	Figures
A	1* T*	○		–	–	On the left of the first intrawell resonance	(223, 197, 164)	Figures 4, 5, 7, 13, 18, 19 and 20
B	1* T*	○		–	–	In lower frequency range than Branch A	(149, 99, 99)	Figure 19
C	1* T*	○		–	–	In higher frequency range than Branch A	(202, 158, 103)	Figures 4, 5, 13, 15, 16, 17, 18, 19 and 20
D	1* T*		○	○		In the first primary resonance	(179, 224, 200)	Figures 4, 5, 7, 18, 19 and 20
E	1* T*		○	○		In the second primary resonance	(255, 229, 142)	Figures 15, 18 and 19
F	1* T*		○	○		Between/across Branch D and Branch E	(143, 191, 255)	Figures 10, 12, 13, 14, 16, 17, 18, 19 and 20
G	1* T*		○		○	In the first primary resonance	(207, 185, 255)	Figures 7, 18 and 19
H	1* T*		○		○	Borders on the left stable region of Branch F	(255, 169, 154)	Figures 10 and 18
I	2* T*		○		○	In the second primary resonance	(255, 194, 255)	Figures 15, 16, 17, 18, 19 and 20
J	2* T*	○		–	–	In the second primary resonance	(178, 239, 255)	Figures 18, 19 and 20
K	3* T*		○	○		Across entire frequency range	(69, 179, 172)	Figures 18, 19 and 20
L	3* T*		○		○	Across entire frequency range	(255, 177, 67)	Figures 18, 19 and 20

Sym. and asym. indicate symmetry and asymmetry of dynamical motion with respect to x = 0. Herein, the period T is defined by 2π/Ω where Ω is the excitation frequency. For comparison purposes, the numerically obtained results are indicated with color codes (rgb) in the figures.

**Figure 4:**
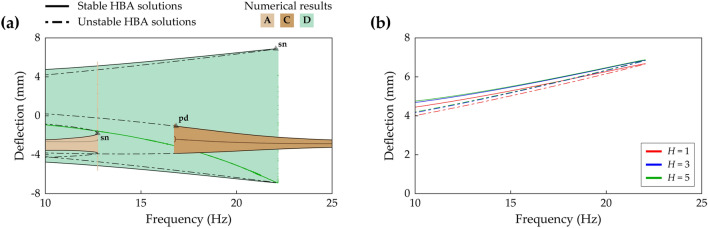
Frequency response and HBA results of the conventional BEH when the base acceleration is 4 m/s^2^. The values of Case A (Table 1) are used. **(a)** Shows sweep responses, stroboscopic points, and HBA solutions of Branch A, C, and D (Table 2). **(b)** Depicts the HBA results of Branch D when the solution truncation order, *H*, is set to be 1, 3, and 5 (Eqs. (6), (7)). Stable and unstable HBA solutions are plotted by the solid line and dot-dashed line, respectively. In **(a)**, the triangle markers indicate the bifurcation points as follows: *sn* saddle-node bifurcation, *pd* period-doubling bifurcation.

**Figure 5:**
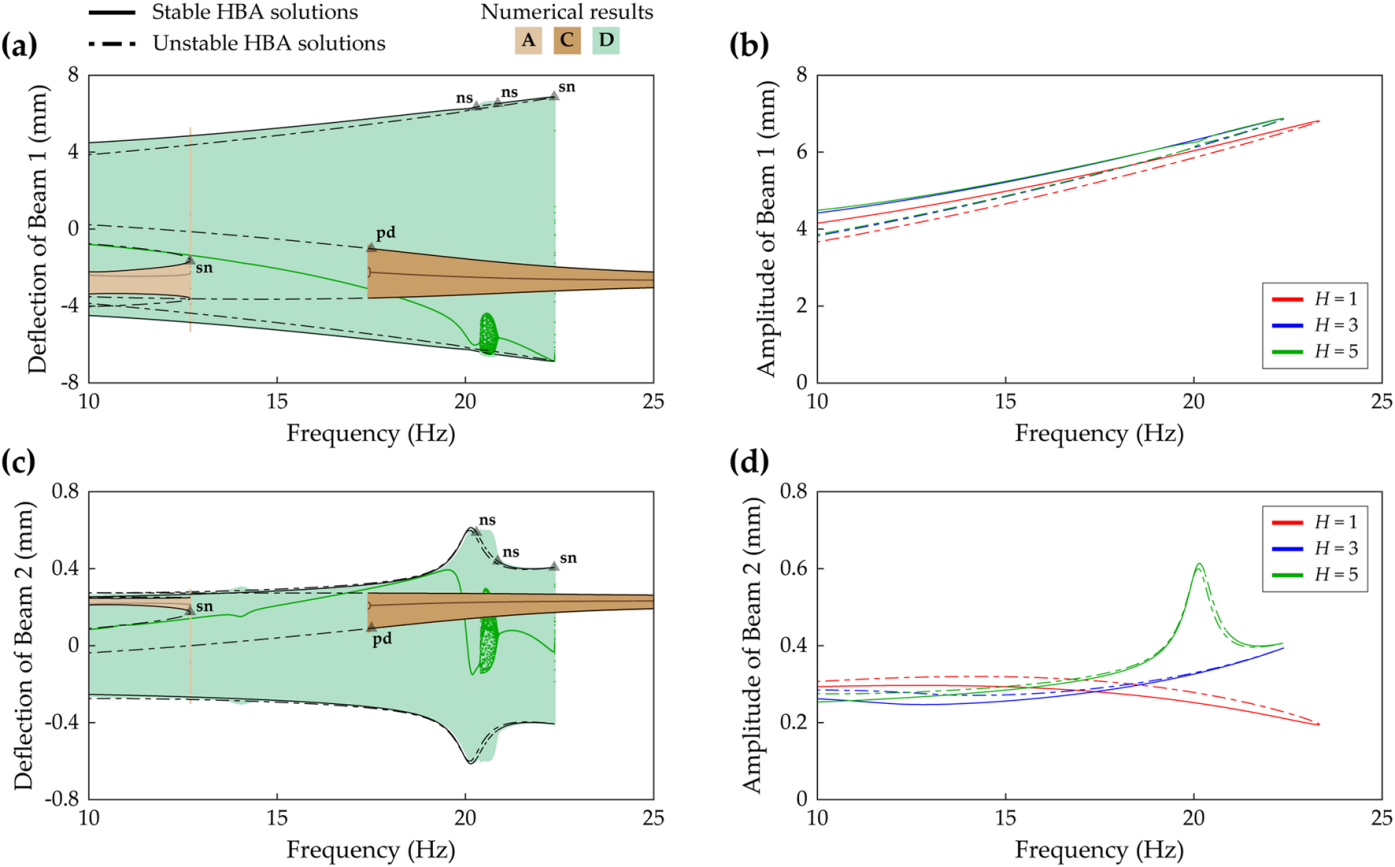
Frequency responses and HBA results of Case B (Table 1) when the base acceleration is 4.4 m/s^2^. The first row **(a,b)** and the second row **(c,d)** are the results of Beam 1 and Beam 2, respectively. The first column **(a,c)** shows sweep responses, stroboscopic points, and HBA solutions of Branch A, C, and D (Table 2). The second column **(b,d)** depicts the HBA results of Branch D when the solution truncation order, *H*, is set to be 1, 3, and 5 (Eqs. (6), (7)). Stable and unstable HBA solutions are plotted by the solid line and dot-dashed line, respectively. In **(a,c)**, the triangle markers indicate the bifurcation points as follows: *sn* saddle-node bifurcation, *pd* period-doubling bifurcation, *ns* Neimark–Sacker bifurcation.

“The first primary resonance” section is mainly focused on resolving the limitations of the single-harmonic solution in the first primary resonance. As shown later, the high-order harmonics should be involved for HBAs at least up to the third-order. “Isolated branch of symmetrical interwell motion” section studies Branch F and Branch H, and parameter study is conducted with respect to beam length ratio. Four regimes according to the beam length ratio are declared. After investigating solution branches in the second primary resonance in “The second primary resonance” section, bridging behavior between the first and the second primary resonances is studied in “Bridging behavior” section. As shown later, Branch F plays a more important role in bridging behavior than period—3* T* solution branches.

### The first primary resonance

It has been reported that in the first primary resonance of 2-DOF MCBEHs, harmonic distortions are not negligible^33^. It implies that HBAs should involve high-order harmonic components. However, for the conventional BEH, the single-harmonic solution has been widely employed for HBAs. This section emphasizes that whereas the primary resonant behavior of the conventional BEH is well described by the first-order truncated solution, this single-harmonic assumption could lead to wrong conclusions for the 2-DOF MCBEHs. In addition, this section reports bifurcation structures of the 2-DOF MCBEHs which are not observed in the conventional BEH.

Figure 4a shows the frequency response of the conventional BEH, which is Case A (beam length ratio = 0), when the base acceleration is 4 m/s^2^. Branch A has a saddle-node bifurcation at which the turning of the branch and discontinuous jump of sweep response occur. Branch C becomes unstable after a period-doubling bifurcation, and the stroboscopic points clearly demonstrate the period-doubling. Branch D has stable period—1* T* interwell solutions until the turning point. Figure 4b demonstrates that for the conventional BEH, the single-harmonic solution is approximately valid for the HBA when compared to the third- or fifth-order truncated solutions. As shown in Fig. 4b, the HBA results by the third- and fifth-order truncations are indistinguishable, and the single-harmonic solution branch also closely matches those two branches. It demonstrates that the first-order truncated solution is approximately suitable to investigate the first primary resonant behavior of the conventional BEH. However, as shown next, this single-harmonic solution is unacceptable for 2-DOF MCBEHs.

Figure 5 provides an example in which the single harmonic assumption in the HBA yields a wrong structure of bifurcations. Figure 5a,c show frequency responses of the 2-DOF MCBEH in Case B (beam length ratio = 0.429) when the base acceleration is 4.4 m/s^2^. The bifurcation structures of Branch A and Branch C are the same as the previous ones in Fig. 4. However, for Branch D, Neimark–Sacker bifurcations occur at 20.3 Hz and 20.85 Hz, which were not observed in the conventional BEH. Figure 5b,d demonstrate the first-order truncation on harmonic balance solution is an oversimplification for Branch D. The single-harmonic solution is obviously different from the high-order truncated solutions especially for Beam 2 as shown in Fig. 5d. The first-order truncation misestimates not only the amplitudes of Branch D, but also the frequency at which the saddle-node bifurcation occurs. Furthermore, the Neimark–Sacker bifurcations are neglected, and the unstable region is regarded as the stable region. Figure 5b,d show that at least third-order truncation is required to estimate the accurate turning point. In addition, Fig. 5d clearly shows that the Neimark–Sacker bifurcations can be obtained by the fifth-order truncated solution.

**Figure 6:**
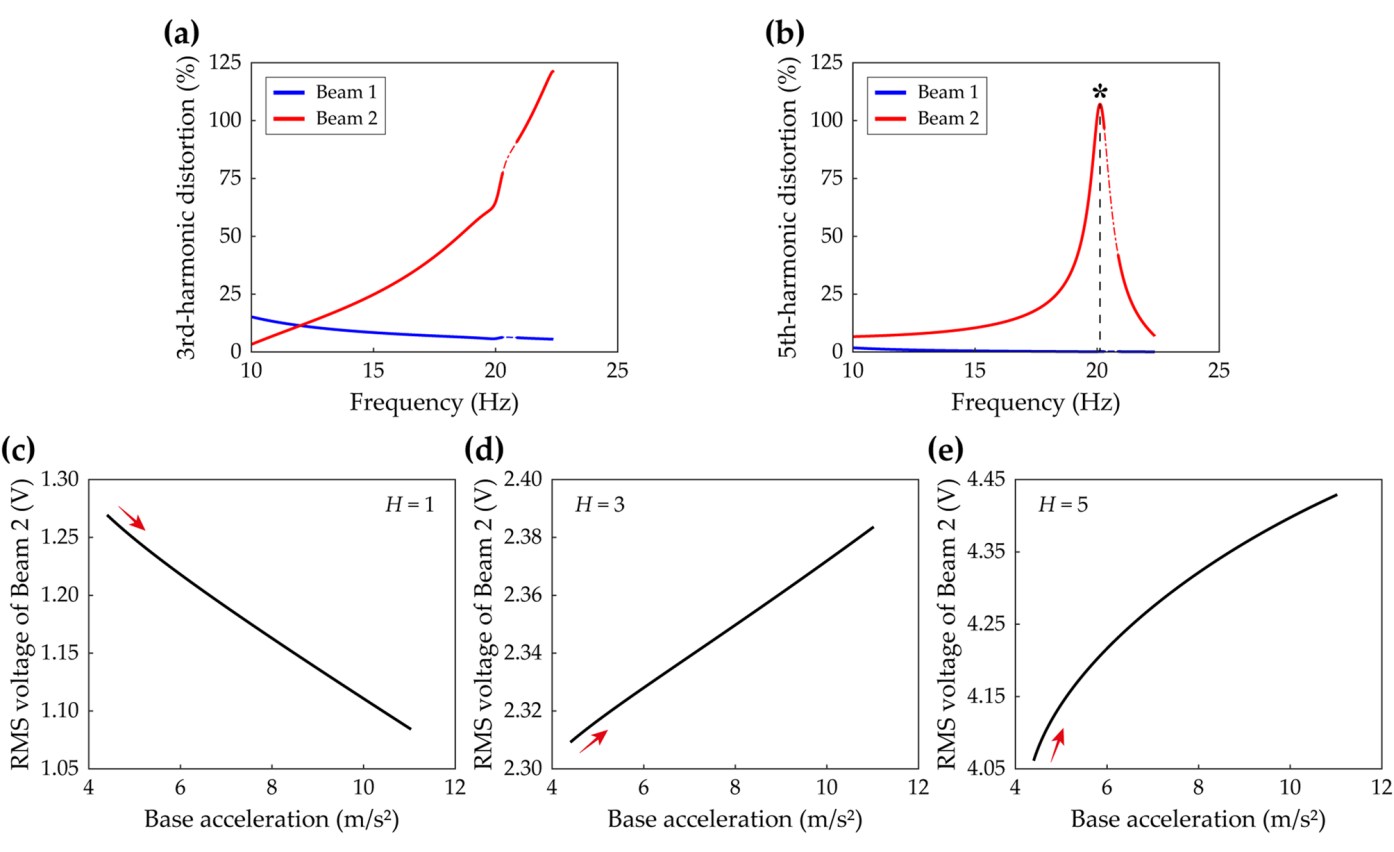
Harmonic distortions and their effects on simulations for root-mean-square (RMS) voltage calculations of Branch D (Table 2) of Beam 2 in Case B (Table 1). The *k*-th harmonic distortion means relative deflection amplitude of the *k*-th order harmonic with respect to the amplitude of the fundamental harmonic. **(a,b)** Show the third- and fifth-harmonic distortions of Branch D in Fig. 5, respectively. In **(a,b),** the dot-dashed line represents unstable solutions. In **(b)**, the asterisk denotes the maximum point of the fifth-harmonic distortion of Beam 2, and the frequency corresponding to the asterisk is used in **(c–e)**. For the simulations of the RMS voltage outputs, the base acceleration is increased from 4.4 m/s^2^, used in Fig. 5, to  11 m/s^2^. **(c–e)** Show the RMS voltage outputs of Beam 2 when the solution truncation order, *H*, is set to be 1, 3, and 5 (Eqs. (6), (7)), respectively. The red arrows indicate whether the RMS voltage output increases or decreases as the base excitation becomes strong.

Moreover, Fig. 6 demonstrates that using the single-harmonic solution could lead to a wrong conclusion about the relationship between root-mean-square (RMS) voltage outputs and base accelerations. Figure 6a,b show the third- and fifth-harmonic distortions of Branch D in Fig. 5, respectively. Herein, the *k*-th harmonic distortion is introduced as

9 $$k{\text{-th harmonic distortion}} = \sqrt {a_{ck}^{2} + a_{sk}^{2} } /\sqrt {a_{c1}^{2} + a_{s1}^{2} } ,$$

which represents the relative deflection amplitude of the *k*-th order harmonic with respect to the amplitude of the fundamental harmonic. As shown in Fig. 6a, the third-harmonic distortion of Beam 2 increases to significant magnitude as the excitation frequency increases while the third-harmonic distortion of Beam 1 stays small. Figure 6b shows that the fifth-harmonic distortion of Beam 1 is negligible. This was suggested previously by Fig. 5b in which the third- and fifth-order truncated solutions of Beam 1 were almost indistinguishable. However, for Beam 2, the local peak of frequency response was estimated by the fifth-order truncated solution as depicted previously in Fig. 5d. Figure 6b, in which Beam 2 has the maximum fifth-harmonic distortion at 20.1 Hz indicated by the asterisk, implies that the fifth-harmonic distortion produces a significant effect on the local peak and the Neimark–Sacker bifurcations in Fig. 5. At the frequency corresponding to the asterisk in Fig. 6b, the relationship between RMS voltage outputs of Beam 2 and base accelerations is investigated according to the truncation order of the HBA solution. As concluded by Lan et al.^32^, Fig. 6c shows that the single-harmonic solution suggests that the stronger excitation leads to the attenuation of harvested voltage. However, because the single-harmonic solution misestimates the response as demonstrated in Fig. 5, this statement is unreliable. In fact, for Branch D of the 2-DOF MCBEH in Case B, the relationship between RMS voltage outputs and base accelerations is proportional when the third- or fifth-order truncated solutions are employed as shown in Fig. 6d,e. As implied in Fig. 5d, the third-order truncated solution underestimated the amplitude of Beam 2 because the fifth-harmonic component was neglected. Likewise, the RMS voltage values in Fig. 6d are underestimated compared to the values in Fig. 6e. Although the third-order truncation yields inaccurate values, it identifies the proportional relationship between RMS voltage outputs and base accelerations.

**Figure 7:**
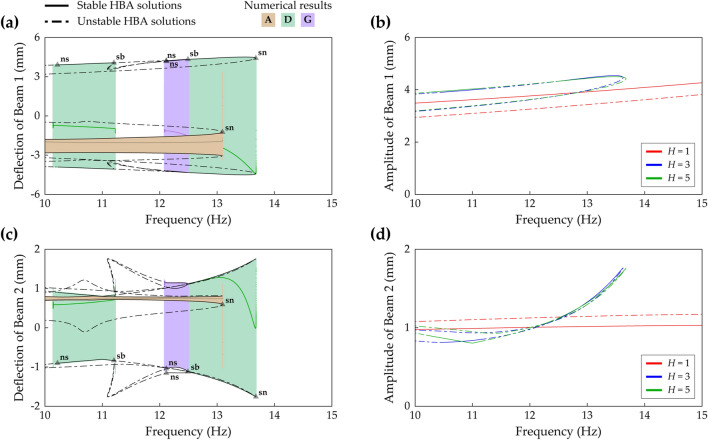
Frequency responses and HBA results of Case C (Table 1) when the base acceleration is 5 m/s^2^. The first row **(a,b)** and the second row **(c,d)** are the results of Beam 1 and Beam 2, respectively. The first column **(a,c)** shows sweep responses, stroboscopic points, and HBA solutions of Branch A, D, and G (Table 2). The second column **(b,d)** depicts the HBA results of Branch D when the solution truncation order, *H*, is set to be 1, 3, and 5 (Eqs. (6), (7)). Stable and unstable HBA solutions are plotted by the solid line and dot-dashed line, respectively. In **(a,c)**, the triangle markers indicate the bifurcation points as follows: *sn* saddle-node bifurcation, *sb* symmetry-breaking bifurcation, *ns* Neimark–Sacker bifurcation.

Figure 7 gives another example in which the single-harmonic approximation leads to wrong results in the first primary resonance. Figure 7a,c show frequency responses of the 2-DOF MCBEH in Case C (beam length ratio = 0.7) when the base acceleration is 5 m/s^2^. Branch A has the same bifurcation structure as the conventional BEH or Case B. However, in Case C, asymmetrical interwell oscillation emerges, and the architecture of the first primary resonance becomes different when compared to Fig. 5a,c. Branch D, period—1* T* symmetrical interwell solution, has a Neimark–Sacker bifurcation at 10.2 Hz, a saddle-node bifurcation at 13.7 Hz, and two symmetry-breaking bifurcations in the middle of Branch D where Branch G is found. When the excitation frequency increases from the left symmetry-breaking bifurcation point at 11.2 Hz, the response shows aperiodic motion after a short region of asymmetrical motion until the excitation frequency reaches the stable region of Branch G. For backward sweep from the right symmetry-breaking bifurcation point at 12.5 Hz, the motion becomes asymmetrical with respect to **x** = **0**. The stability of Branch G, period—1* T* asymmetrical interwell solution, is changed at a Neimark–Sacker bifurcation at 12.1 Hz. In the region where Branch G is unstable, interwell chaotic attractors and period—1* T* intrawell motion (Branch A) coexist. Because this asymmetrical interwell motion cannot be described by only the fundamental harmonic, an HBA with the single-harmonic solution would ignore Branch G, thereby leading to a wrong architecture of frequency responses. Furthermore, Fig. 7b,d clearly demonstrate the limitations of the single-harmonic solution for Branch D. Whereas the third-order truncated solution matches approximately the fifth-order truncated solution, the first-order truncated solution yields the obviously wrong result.

**Figure 8:**
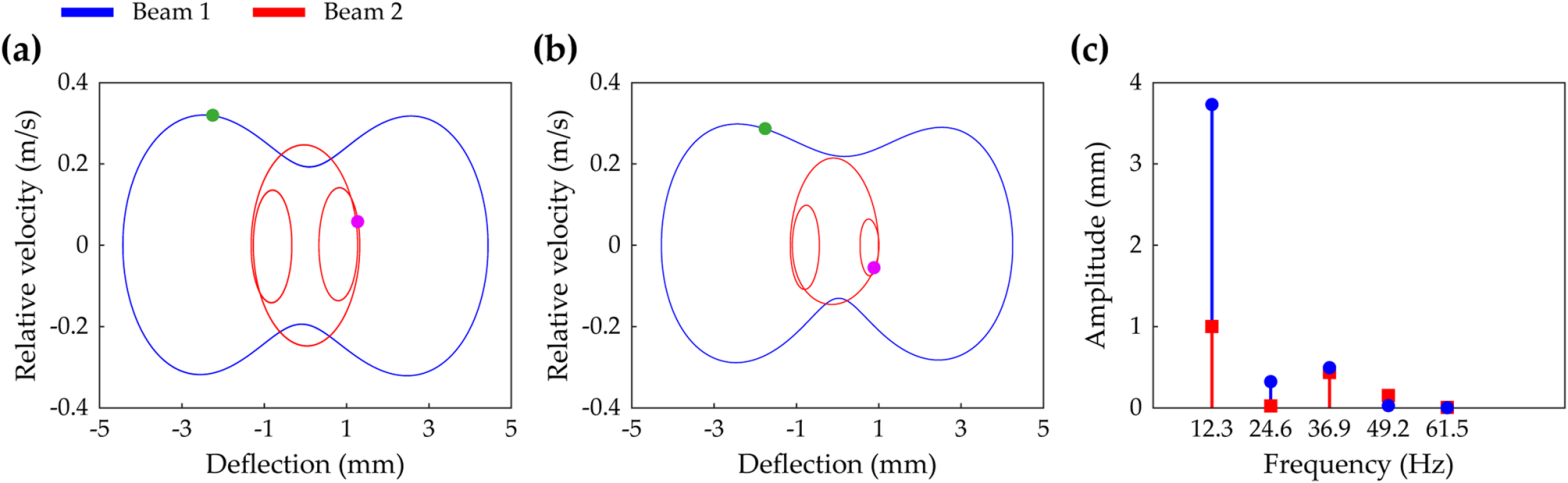
The steady-state interwell motion of the 2-DOF MCBEH with the same conditions of Fig. 7. **(a,b)** Show the phase portraits and stroboscopic points of Branch D (symmetrical) at 13 Hz and Branch G (asymmetrical) at 12.3 Hz, respectively. **(c)** Is the corresponding FFT result of **(b)**.

Steady-state phase portraits of Branch D at 13 Hz and Branch G at 12.3 Hz in Fig. 7 are shown in Fig. 8a,b, respectively. By comparing Fig. 8a,b, the asymmetry of Branch G is easily noticed. Moreover, as shown in Fig. 8c, the FFT result, corresponding to Fig. 8b, shows that the magnitudes of the high-order harmonic components are significant. It implies that the assumed solution form for an HBA, Eqs. (6) and (7), should involve these high-order terms for the HBA to obtain Branch G. Especially, the even harmonics are essential to describe the asymmetry of Branch G.

**Figure 9:**
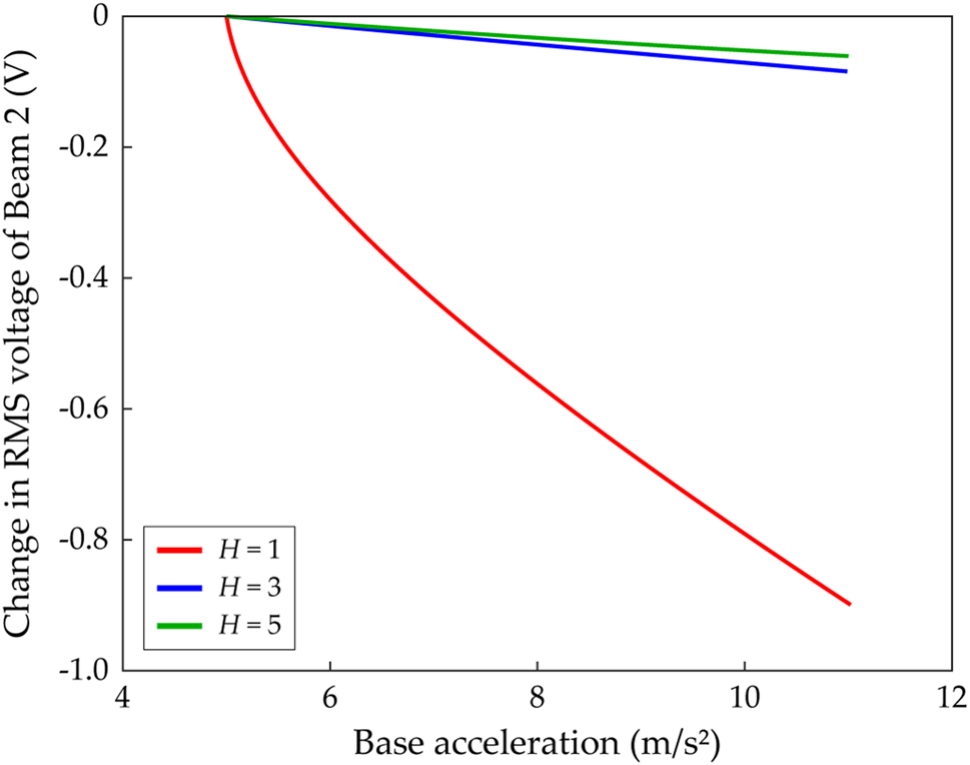
Change in RMS voltage of Branch D (Table 2) of Beam 2 in Case C (Table 1) according to the base acceleration. The base acceleration is increased from 5 m/s^2^, used in Fig. 7, to 11 m/s^2^. The red, blue, green lines are obtained when the solution truncation order, *H*, is set to be 1, 3, and 5 (Eqs. (6), (7)), respectively. For the third- and fifth-order truncated solutions, the excitation frequency is 11 Hz. For the first-order truncated solution, the excitation frequency is 24.9 Hz. For these excitation frequencies, the HBA solutions are stable (refer to Fig. 7b,d).

Figure 9 depicts changes in RMS voltage outputs of Branch D of Beam 2 in Case C as base acceleration increases when the responses are estimated by the first-, third-, and fifth-order truncated solutions. For the single-harmonic solution, the excitation frequency is set to be 24.9 Hz. Figure 9 shows that when the HBA solution is truncated up to the first order, the RMS voltage outputs of Beam 2 decreases noticeably for the stronger excitation. However, as demonstrated in Fig. 7, the HBA solution should be truncated up to at least third order to describe the response accurately. When the high-order truncated solution forms are employed, decrements of RMS voltage outputs of Beam 2 at 11 Hz for the higher base accelerations are negligible when compared to the overestimated changes obtained by the single-harmonic solution. Lan et al. concluded that based on the single-harmonic solution form, these decrements always occur and limit the harvesting performance of 2-DOF MCBEHs^32^. In fact, when the high-order truncated solutions are employed in the HBAs, those decrements could be wrong estimates as demonstrated in Fig. 6 or have negligible magnitudes as compared in Fig. 9.

### Isolated branch of symmetrical interwell motion

The present paper firstly reports the dynamics of Branch F, the period—1* T* large orbit solution branch between or across the first and the second primary resonances. Because this dynamical behavior is a distinctive characteristic for 2-DOF MCBEHs, Branch F should be revealed by the HBA and discussed in terms of energy harvesting performance.

**Figure 10:**
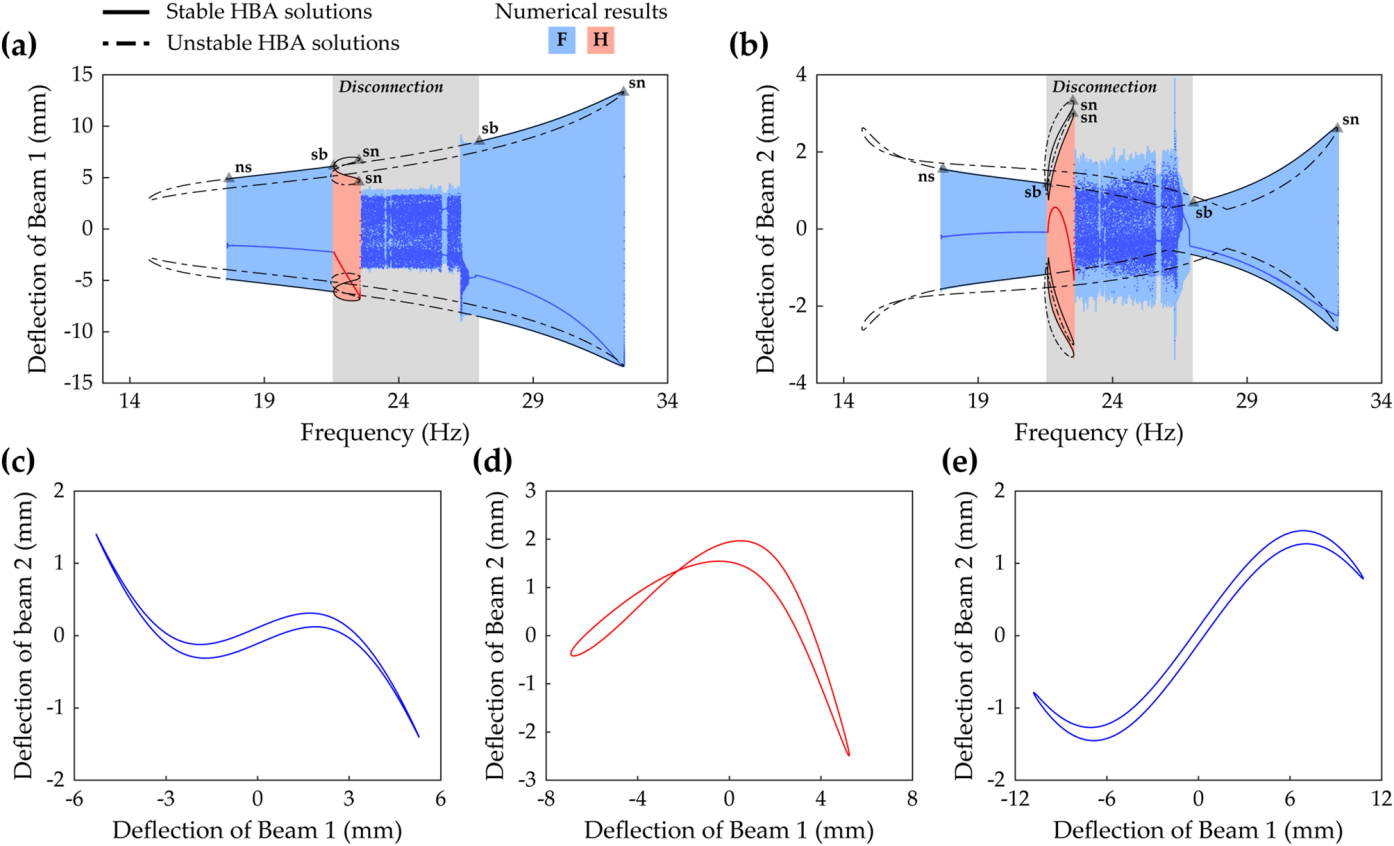
Frequency responses and *w*_1_–*w*_2_ plots of Branch F and Branch H (Table 2) in Case C (Table 1) when the base acceleration is 10 m/s^2^. **(a,b)** Show sweep responses, stroboscopic points, and HBA solutions of Beam 1 and Beam 2, respectively. Stable and unstable HBA solutions are plotted by the solid line and dot-dashed line, respectively. The region in gray color represents the disconnected frequency bandwidth of Branch F due to instabilities. **(c–e)** Are the *w*_1_–*w*_2_ plots of Branch F at 19 Hz, Branch H at 22 Hz, and Branch F at 30 Hz, respectively. In **(a,b)**, the triangle markers indicate the bifurcation points as follows: *sn* saddle-node bifurcation, *sb* symmetry-breaking bifurcation, *ns* Neimark–Sacker bifurcation.

Figure 10 shows the architecture in frequency response space of Branch F and Branch H in Case C (beam length ratio = 0.7) when the base acceleration is 10 m/s^2^. Branch H, period—1* T* interwell motion asymmetrical with respect to **x** = **0**, exists nearby the left symmetry-breaking bifurcation point of Branch F as shown in Fig. 10a,b. In the left stable region of Branch F, the instabilities are caused by a Neimark–Sacker bifurcation at 17.7 Hz and the symmetry-breaking bifurcation at 21.6 Hz. In the right stable region, two bifurcations are observed; a symmetry-breaking bifurcation at 27.0 Hz and a saddle-node bifurcation at 32.4 Hz. A discontinuous jump occurs at the saddle-node bifurcation, and for backward sweep through the right symmetry-breaking bifurcation, the response becomes aperiodic shortly after asymmetrical motion. Branch H borders on the left stable region of Branch F and the left symmetry-breaking bifurcation point. On the right boundary of Branch H, the solution branch is folded at a saddle-node bifurcation which occurs at 22.5 Hz. Because none of the stable periodic orbit exists beyond this saddle-node bifurcation (refer to Fig. 18 which depicts all solution branches), the system shows chaotic motion as shown in Fig. 10a,b. The shaded region in Fig. 10a,b indicates a disconnection between the left and right stable regions of Branch F. If Branch F is utilized for energy harvesting, this disconnected frequency bandwidth should be mentioned as a drawback to broadband performance. However, as demonstrated later, this limitation is overcome by designing the beam length ratio. Figure 10c–e show *w*_1_–*w*_2_ plots of Branch F at 19 Hz, Branch H at 22 Hz, and Branch F at 30 Hz, respectively. As shown in Fig. 10c, which belongs to the left stable region of Branch F, the motion of Beam 1 and Beam 2 is more dominated by the out-of-phase mode regime than the in-phase mode regime because the excitation frequency is close to the first primary resonance^16^. Figure 10d shows that the motion of Branch H is asymmetrical with respect to **x** = **0** and implies that the motion belongs to neither of the regimes. Figure 10e shows that when the excitation frequency is close to the second primary resonance in the higher frequency range, the phase-dependent dynamics is dominated by the in-phase mode regime^16^.

**Figure 11:**
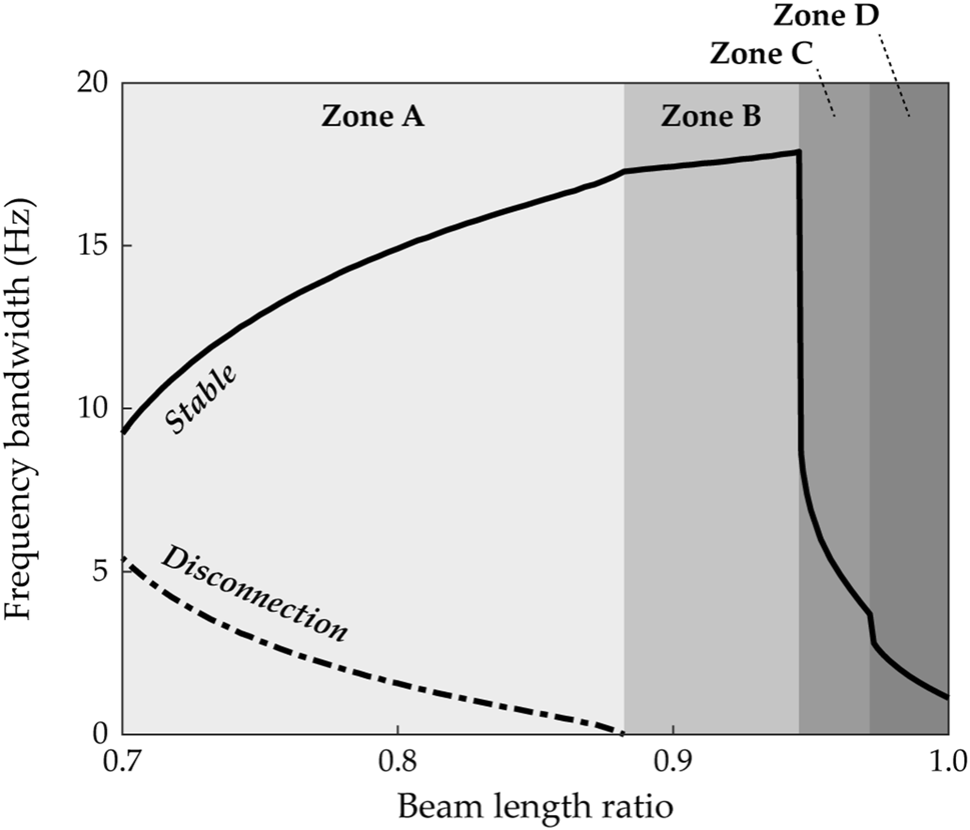
Parameter study on the frequency bandwidth of Branch F (Table 2) with respect to beam length ratio when the base acceleration is 10 m/s^2^. The solid line represents the frequency bandwidth of the stable HBA solution. The dot-dashed line indicates the frequency bandwidth of the unstable region between two stable regions (refer to Fig. 10a,b). According to the parameter study results, four zones can be categorized. When the beam length ratio belongs to Zone A, a disconnected region exists. However, in Zone B, the stable solution branch is not interrupted by instability. When the beam length ratio reaches Zone C, the stable bandwidth decreases rapidly. The range after another sudden bandwidth drop is denoted by Zone D.

Figure 11 suggests that the architecture of Branch F can be categorized into four zones according to beam length ratio. The disconnected frequency bandwidth, indicated by the shaded region in Fig. 10a,b, and the stable frequency bandwidth of Branch F is obtained as the beam length ratio is increased from 0.7 to 1. The base acceleration is chosen as 10 m/s^2^ and the separation distance is set to be 600-μm ahead of the pitchfork bifurcation point of static equilibrium for each beam length ratio. The architecture in Fig. 10 belongs to Zone A in Fig. 11. Branch F in Zone A has the disconnection by which broadband performance degradation is caused. In Zone A, it is noteworthy that as the beam length ratio increases, stable frequency bandwidth increases, but disconnected frequency bandwidth decreases. When the beam length ratio reaches Zone B, the disconnection disappears, and the stable solution branch is no longer interrupted by instability. As the beam length ratio increases further, the working bandwidth for energy harvesting becomes suddenly narrower. Zone C indicates the system conditions after this scenario occurs as illustrated in Fig. 11. Subsequently, another sudden drop in performance bandwidth is observed, and afterward the region is named Zone D. Considering the above-mentioned observations, it is concluded that Branch F in Zone B is promising for energy harvesting by 2-DOF MCBEHs.

**Figure 12:**
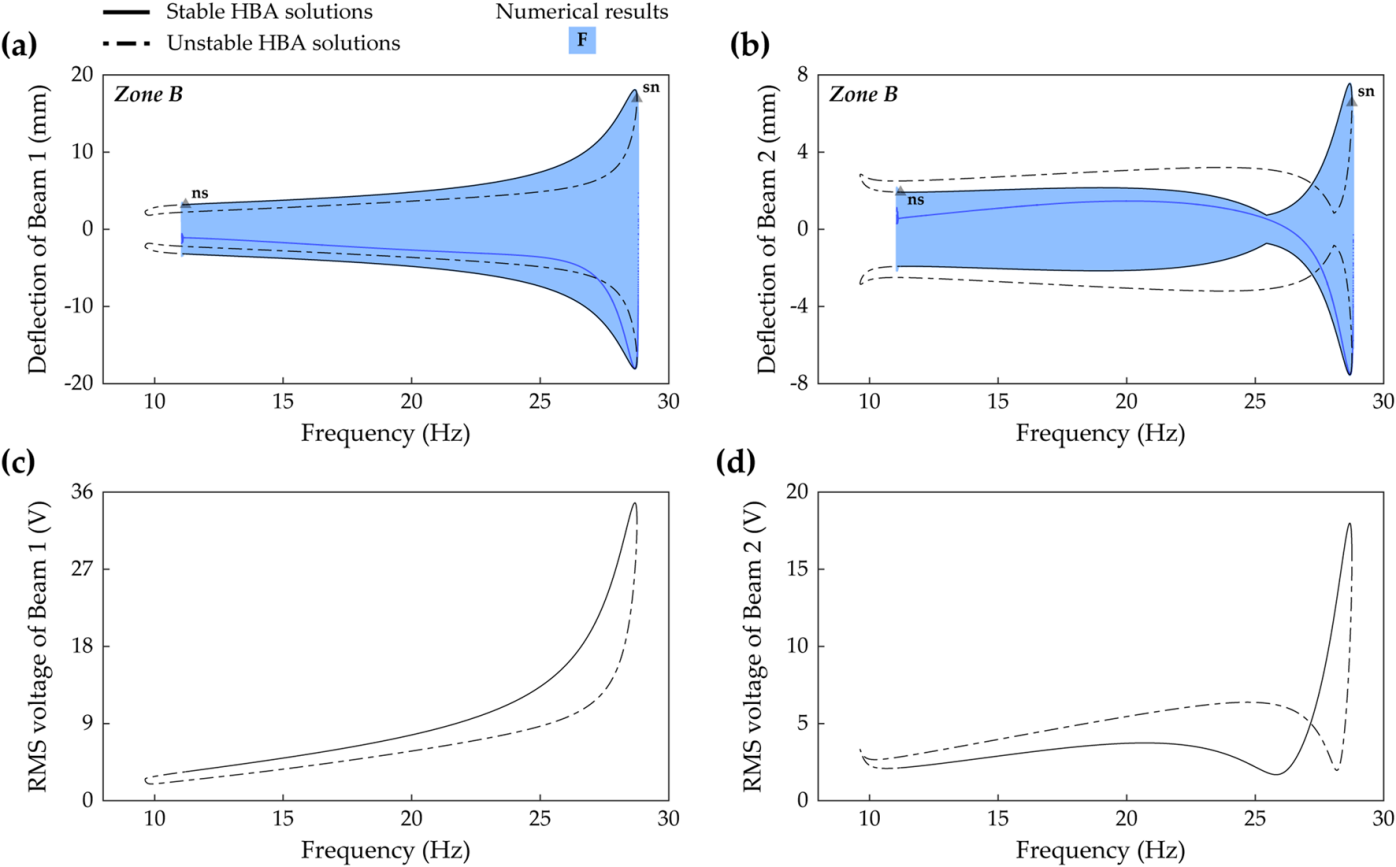
Frequency responses and RMS voltage outputs of Branch F (Table 2) in Case D (Table 1) when the base acceleration is 10 m/s^2^. **(a,b)** Show sweep responses, stroboscopic points, and HBA solutions of Beam 1 and Beam 2, respectively. **(c,d)** Depict RMS voltage outputs of Beam 1 and Beam 2, respectively. Stable and unstable HBA solutions are plotted by the solid line and dot-dashed line, respectively. In **(a,b)**, *Zone B* means that the branch belongs to Zone B in Fig. 11, and the triangle markers indicate the bifurcation points as follows: *sn* saddle-node bifurcation, *ns* Neimark–Sacker bifurcation.

Case D (beam length ratio = 0.914) belongs to Zone B, and in this case, Branch F in frequency response space is shown in Fig. 12a,b. A Neimark–Sacker bifurcation and a saddle-node bifurcation exist at 11.2 Hz and 28.8 Hz, respectively. The discontinuous jump occurs at the saddle-node bifurcation on the right of Branch F, and on the left side after the Neimark–Sacker bifurcation, the unstable solutions extend until turning to the right. These are the same characteristics as Branch F in Zone A as shown in Fig. 10a,b. However, Branch F in Fig. 12a,b does not have a disconnection by symmetry-breaking bifurcations in the middle of the solution branch, and accordingly, Branch H is not observed. When broadband performance is considered, this continuously stable architecture of Branch F is superior to the previous architecture in which the stable regions were separated by the instabilities. Figure 12c,d show the associated RMS voltage outputs of Beam 1 and Beam 2, respectively. For Beam 1, the RMS voltage is identified as a monotonically increasing function of excitation frequency except for the small range before the saddle-node bifurcation. Harvested power by Beam 1 becomes magnified as the excitation frequency increases. In contrast, the RMS voltage of Beam 2 is not a monotonic function of excitation frequency. The local minimum point is observed at 25.8 Hz as shown in Fig. 12d, and the neighborhood of this local minimum might be regarded as a drawback. Nevertheless, because Branch F extends across the long range of excitation frequencies with interwell motion, this solution branch is promising for enhanced broadband performance.

**Figure 13:**
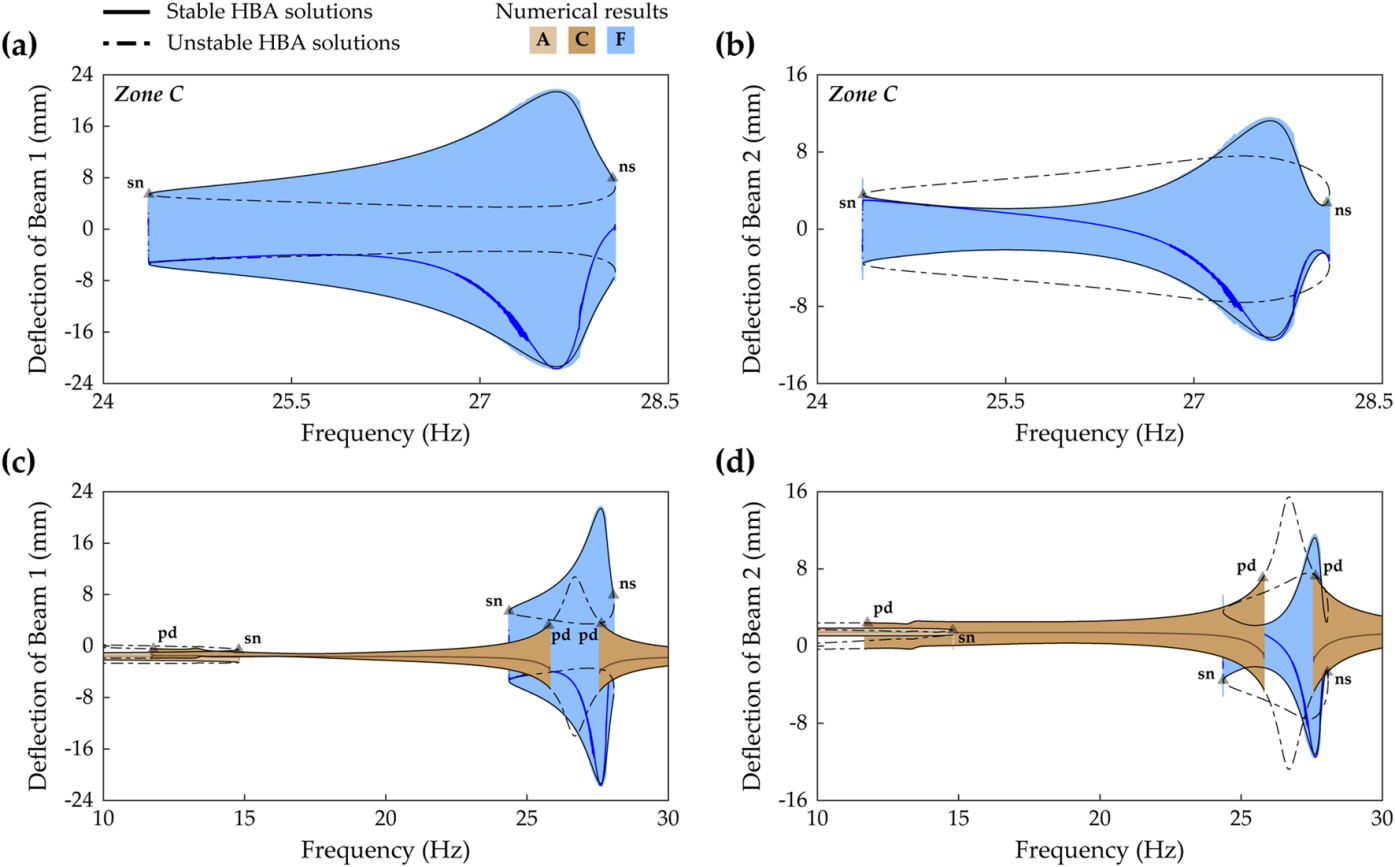
Frequency responses of Branch A, C, and F (Table 2) in Case E (Table 1) when the base acceleration is 10 m/s^2^. **(a,b)** Show sweep responses, stroboscopic points, and HBA solutions of Beam 1 and Beam 2, respectively. Stable and unstable HBA solutions are plotted by the solid line and dot-dashed line, respectively. In **(a,b)**, *Zone C* means that the branch belongs to Zone C in Fig. 11. The triangle markers indicate the bifurcation points as follows: *sn* saddle-node bifurcation, *pd* period-doubling bifurcation, *ns* Neimark–Sacker bifurcation.

Figure 13a,b show Branch F in Case E (beam length ratio = 0.971) which belongs to Zone C. Branch F has a saddle-node bifurcation at 24.4 Hz where discontinuous jump occurs and a Neimark–Sacker bifurcation at 28.1 Hz. There is a region from 26.8 to 27.4 Hz in which oscillations are quasi-periodic due to higher-order effects, and these instabilities were not captured by the HBA performed in this study. It is notable that the bifurcation structure is different from the previous one in Fig. 12a,b. On the right end of Branch F, the solution branch loses stability at the Neimark–Sacker bifurcation point, and the unstable branch bulges slightly. When the forward sweep is conducted from Branch F, it is observed that quasi-periodic motion appears within a short range after the Neimark–Sacker bifurcation, and subsequently, a discontinuous jump occurs. As shown later in Fig. 16, the right bifurcation is identified as a saddle-node bifurcation if the base acceleration is 9 m/s^2^. Branch F has the turning point at the saddle-node bifurcation on the left end of the branch, and there is no extended unstable branch toward the left side which was observed in the previous Branch F with the Neimar–Sacker bifurcation in Fig. 12a,b. As demonstrated later, this difference results in different bridging behavior, a connection between Branch F and the first primary resonant interwell branch, when the base acceleration is high enough.

Figure 13c,d show frequency responses of period—1* T* oscillations from 10 to 30 Hz in Case E for comparisons to Fig. 12a,b. As demonstrated in Fig. 11, the harvesting bandwidth of Branch F in Zone C was drastically narrower than the bandwidth in Zone B. Herein, it is identified that Branch F in Zone C is placed locally around the second primary intrawell resonance. In this resonance, Branch C, period—1* T* intrawell oscillation, loses stabilities by two period-doubling bifurcations at 25.8 Hz and 27.6 Hz. Although the solution branch is unstable, the peak at 26.7 Hz confirms the second primary intrawell resonant area. When comparing Fig. 13c,d with Fig. 12a,b, it is noticed that the broadband performance of Branch F in Zone C is inferior to Branch F in Zone B.

**Figure 14:**
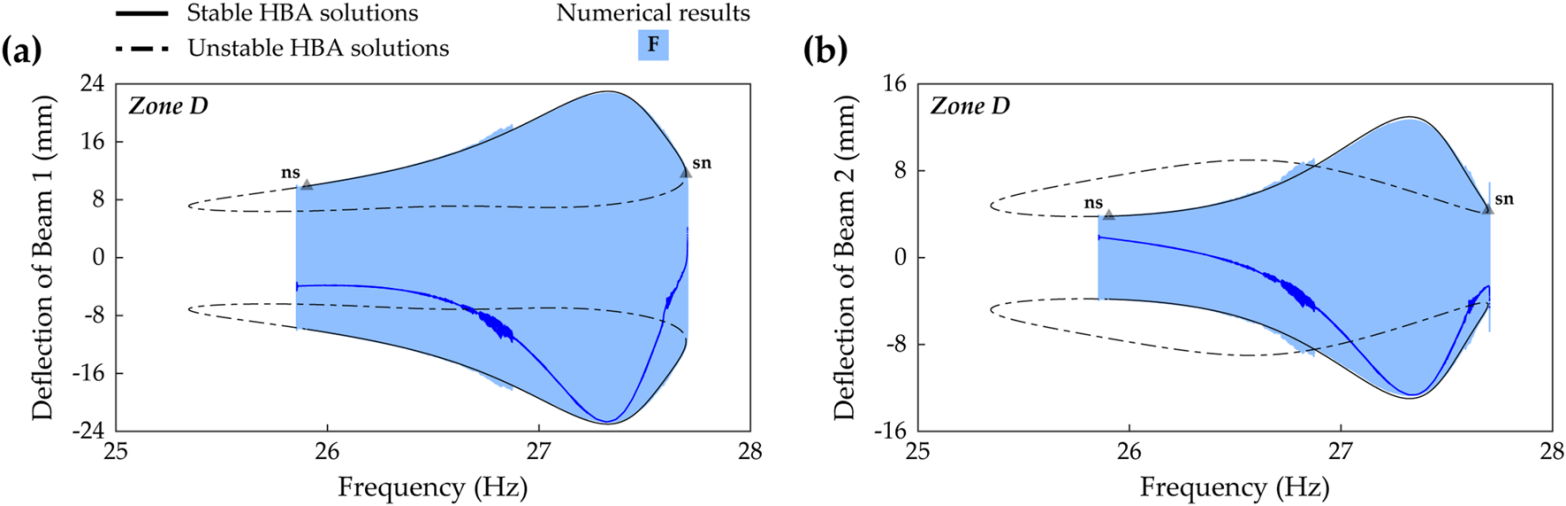
Frequency responses of Branch F (Table 2) in Case F (Table 1) when the base acceleration is 10 m/s^2^. **(a,b)** Show sweep responses, stroboscopic points, and HBA solutions of Beam 1 and Beam 2, respectively. Stable and unstable HBA solutions are plotted by the solid line and dot-dashed line, respectively. *Zone D* means that the branch belongs to Zone D in Fig. 11. The triangle markers indicate the bifurcation points as follows: *sn* saddle-node bifurcation, *ns* Neimark–Sacker bifurcation.

Figure 14 shows frequency responses of Branch F in Case F (beam length ratio = 0.986) which belongs to Zone D. The distinguishing difference in the branch structure from Zone C is the left unstable tail after the left bifurcation point. In Zone C previously demonstrated in Fig. 13, there was the saddle-node bifurcation at the left turning point. However, the branch architecture in Zone D has a Neimark–Sacker bifurcation on the left and unstable solutions which persist to the left turning point. Herein, it is identified that the underlying cause of the sudden bandwidth degradation, observed in Fig. 11, is the left unstable tail after the Neimark–Sacker bifurcation shown in Fig. 14. In Fig. 14, the Neimark–Sacker bifurcation is observed at 25.9 Hz, and the saddle-node bifurcation is located in the right turning point at 27.7 Hz. In addition, aperiodic oscillations in the middle of Branch F due to higher-order effects are also observed like the phenomenon in Zone C shown in Fig. 13a,b.

### The second primary resonance

The second primary resonance is the unique characteristic of 2-DOF MCBEHs because it does not exist in the conventional BEH. The dynamical behavior in this resonance has been identified by the phase-dependent dynamics^16^. The present study applies the HBA and reveals all coexisting solution branches which have not been reported before^32^. In addition, bifurcation structures are also investigated.

**Figure 15:**
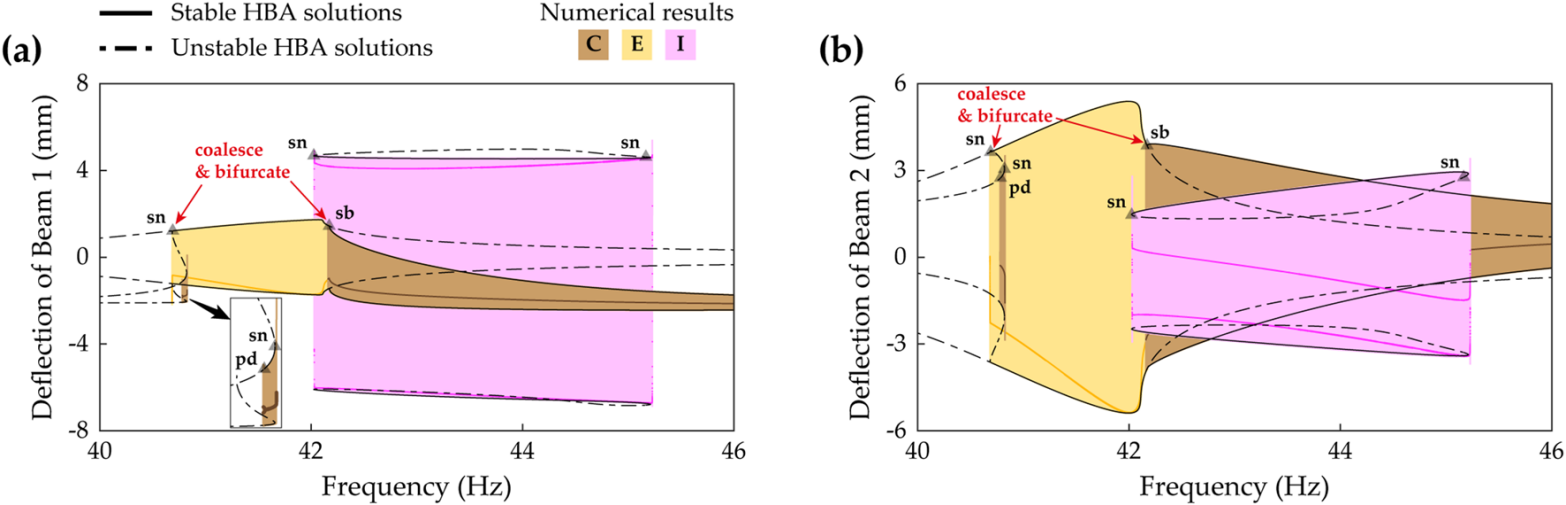
Frequency responses of Branch C, E, and I (Table 2) in Case C (Table 1) when the base acceleration is 9 m/s^2^. **(a,b)** Show sweep responses, stroboscopic points, and HBA solutions of Beam 1 and Beam 2, respectively. The inset in **(a)** displays an enlarged picture to clearly indicate the bifurcation points. The red arrows indicate where Branch C and Branch E coalesce and bifurcate. Stable and unstable HBA solutions are plotted by the solid line and dot-dashed line, respectively. The triangle markers indicate the bifurcation points as follows: *sn* saddle-node bifurcation, *sb* symmetry-breaking bifurcation, *pd* period-doubling bifurcation. The red arrows indicate where Branch C and Branch E coalesce and bifurcate.

Figure 15 shows frequency responses in the second primary resonance in Case C (beam length ratio = 0.7) when the base acceleration is 9 m/s^2^. The small left stable region of Branch C has two bifurcations; a period-doubling bifurcation at 40.78 Hz and a saddle-node bifurcation at 40.82 Hz. After the period-doubling bifurcation, backward sweep shows period—2* T* subharmonic response (refer to Fig. 18). Forward sweep triggers discontinuous jump to the interwell oscillation branch at the saddle-node bifurcation. Along the right stable region of Branch C, backward sweep enters Branch E at a symmetry-breaking bifurcation point at 42.2 Hz. Subsequently, Branch E becomes unstable after a saddle-node bifurcation at 40.7 Hz. These bifurcation structures and dynamical behaviors of Branch C and Branch E have been studied in Ref.^16^. By conducting the HBA here, it is identified that Branch C and Branch E coalesce and bifurcate at the saddle-node bifurcation point and the symmetry-breaking bifurcation point as shown in Fig. 15. Furthermore, the HBA obtains Branch I, period—2* T* asymmetrical interwell oscillation, and this branch is firstly reported in this paper. Because Branch I is isolated in a high-energy orbit, sweep analysis along other solution branches is not allowed to capture Branch I. Two turning points exist in Branch I at 42.0 Hz and 45.2 Hz where discontinuous jump phenomena are observed.

**Figure 16:**
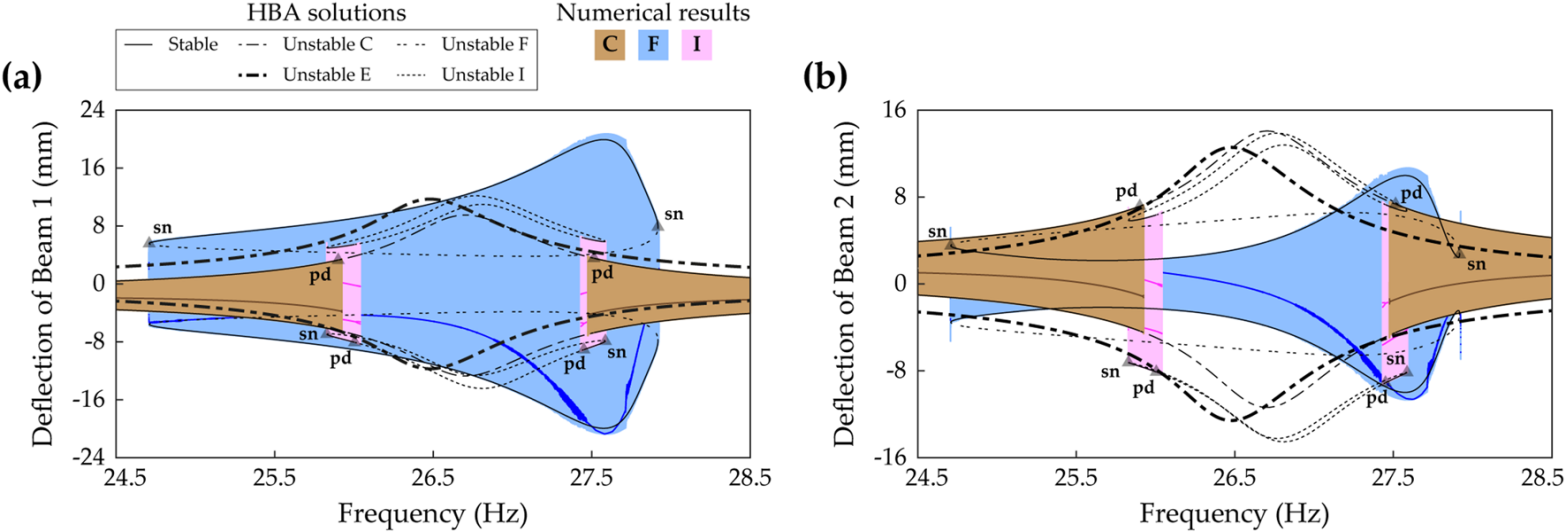
Frequency responses of Branch C, E, F, and I (Table 2) in Case E (Table 1) when the base acceleration is 9 m/s^2^. **(a,b)** Show sweep responses, stroboscopic points, and HBA solutions of Beam 1 and Beam 2, respectively. Because Branch E is unstable in Case E, only HBA results are plotted. The solid line represents stable HBA solutions, and other lines indicate unstable HBA solutions of each branch as follows: dot-dashed line—Branch C, bold dot-dashed line—Branch E, double-dotted line—Branch F, dotted line—Branch I. The triangle markers indicate the bifurcation points as follows: *sn* saddle-node bifurcation, *pd* period-doubling bifurcation.

Figure 16 shows frequency responses in the second primary resonance in Case E (beam length ratio = 0.971) when the base acceleration is 9 m/s^2^. Branch C is unstable around its primary resonant peak, and two period-doubling bifurcations occur at 25.9 Hz and 27.5 Hz. This bifurcation structure is different from the previous one in Fig. 15. In Fig. 15, Branch C coalesced into Branch E at two bifurcation points. In contrast, Fig. 16 shows that Branch E is separated from Branch C and accordingly, coalescing is allowed nowhere. Furthermore, the stability analysis confirms that no stable solution in Branch E is observed. Meanwhile, Branch I coexists in the intrawell resonant range of Branch C. This period—2* T* asymmetrical interwell orbit has two stable regions on the left and right, and the bifurcation structure of the left region is mirror-symmetrical to the one of the right region. The left stable region has a saddle-node bifurcation at 25.8 Hz and a period-doubling bifurcation at 26.0 Hz, and the right region has a saddle-node bifurcation at 27.6 Hz and a period-doubling bifurcation at 27.45 Hz. Moreover, Branch F also coexists, and in this case as demonstrated in the previous section, Branch F belongs to Zone C with a saddle-node bifurcation at the left turning point. The left and right saddle-node bifurcations are found at 24.7 Hz and 27.9 Hz, respectively.

**Figure 17:**
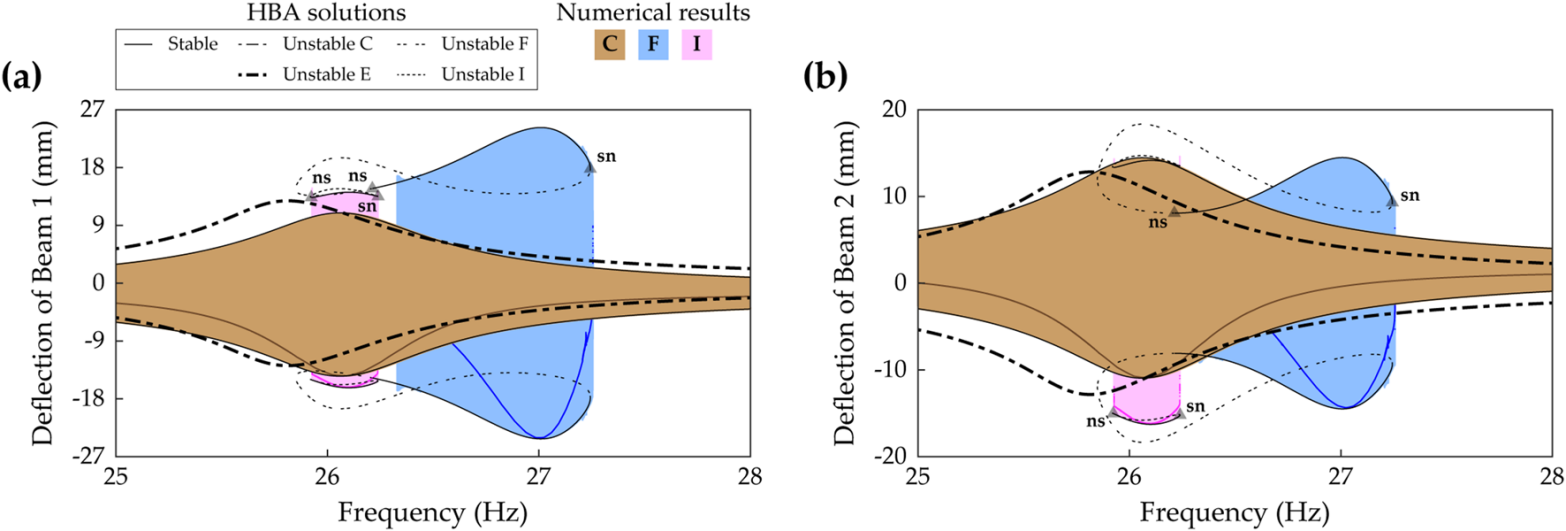
Frequency responses of Branch C, E, F, and I (Table 2) in Case G (Table 1) when the base acceleration is 9 m/s^2^. **(a,b)** Show sweep responses, stroboscopic points, and HBA solutions of Beam 1 and Beam 2, respectively. Because Branch E is unstable in Case G, only HBA results are plotted. The solid line represents stable HBA solutions, and other lines indicate unstable HBA solutions of each branch as follows: dot-dashed line—Branch C, bold dot-dashed line—Branch E, double-dotted line—Branch F, dotted line—Branch I. The triangle markers indicate the bifurcation points as follows: *sn* saddle-node bifurcation, *ns* Neimark–Sacker bifurcation.

Figure 17 shows frequency responses in the second primary resonance in Case G (beam length ratio = 1) when the base acceleration is 9 m/s^2^. In this case, Branch C is stable, and in the region of the intrawell resonance of Branch C, Branch I coexists. Whereas Branch I in Case E was unstable around the intrawell resonant peak as shown previously in Fig. 16, Branch I in Case G is stable as demonstrated in Fig. 17. A Neimark–Sacker bifurcation and a saddle-node bifurcation are found at 25.9 Hz and 26.2 Hz, respectively. For forward sweep in Branch I, a discontinuous jump occurs at the saddle-node bifurcation. For backward sweep, quasi-periodic motion is observed after the Neimark–Sacker bifurcation within the very short range of excitation frequencies, and subsequently, a discontinuous jump is triggered. For Branch F which belongs to Zone D in Fig. 11, a Neimark–Sacker bifurcation at 26.2 Hz and an unstable tail are observed on the left of the branch. On the right, a saddle-node bifurcation is obtained at 27.2 Hz with a discontinuous jump phenomenon. When the architecture of the frequency responses is compared to Fig. 16, it is notable that the isolated high-energy orbits, Branch F and Branch I, are hardly obtained by sweep analysis along Branch C. It suggests that for Case G (beam length ratio = 1), an HBA is essentially required to reveal the dynamical behaviors in the second primary resonance.

### Bridging behavior

It has been believed that the nonlinear behavior of 2-DOF MCBEHs can be utilized to build a bridge between the first primary resonance and the second primary resonance in order to enhance broadband energy harvesting performance^17^. In the previous literature^17^, the direct numerical integration was applied to show the enhanced broadband performance in frequency response space. However, this way is limited to reveal coexisting solutions and regions in which periodic motion is absent. In this regard, the HBA is necessary to disclose the clear, specific configuration of the frequency response. This section reveals three frequency response configurations and reports bridging phenomena studied by the HBA.

**Figure 18:**
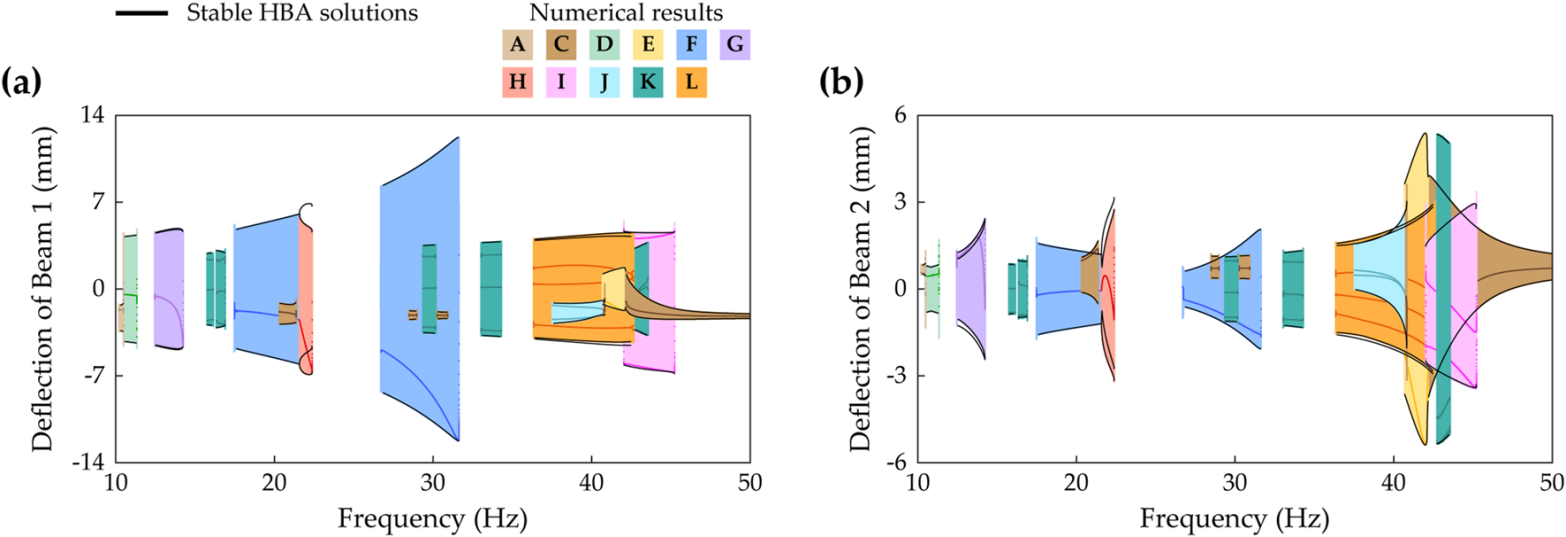
Frequency responses of all periodic solution branches in Case C (Table 1) when the base acceleration is 9 m/s^2^. Refer to Table 2 for branch categorization. **(a,b)** Show sweep responses, stroboscopic points, and HBA solutions of Beam 1 and Beam 2, respectively. Unstable HBA solutions are suppressed in the figure for simplicity.

Figure 18 shows frequency responses of every solution branch in Case C (beam length ratio = 0.7) obtained by the proposed HBA. The base acceleration is chosen as 9 m/s^2^ to make high-energy orbits developed. In the first primary resonant area in the range of low frequencies, the architecture explained in Fig. 7 appears in Fig. 18. Branch A is the period—1* T* intrawell oscillation which produces low energy output. Branch D and Branch G are the period—1* T* interwell oscillations which are symmetrical and asymmetrical with respect to **x** = **0**, respectively. The second primary resonant branches, investigated in Fig. 15, are shown in the region of high frequencies. Branch C, the period—1* T* intrawell oscillation, coalesces into Branch E, the period—1* T* interwell oscillation in the in-phase mode regime. Branch I, the period—2* T* asymmetrical interwell oscillation newly reported in “The second primary resonance” section, coexists with Branch C and Branch E. Besides, the multiple periodic motions are also depicted, and the stroboscopic points, synchronized with the excitation period *T*, imply period—2* T* or —3* T* motions. Branch J is the period—2* T* subharmonic intrawell oscillation with low energy output. This branch starts from the period-doubling bifurcation of Branch C indicated in Fig. 15. Branch K and Branch L are the period—3* T* interwell motions which are symmetrical and asymmetrical with respect to **x** = **0**, respectively. In the second primary resonance, these period—3* T* branches coexist with Branch C, E, I, and J. The HBA and associated sweep results demonstrate that the dynamical behavior in the second primary resonant area is very complicated and that the HBA is useful to study the rich dynamics here. When it comes to Branch K, symmetrical period—3* T* stable solutions emerge across the entire frequency range. However, this long branch is interrupted by many instabilities, thereby leading to chaotic oscillation or other types of periodic oscillation. It suggests that the period—3* T* branch is inappropriate to bridge harvesting bandwidth between the first and second primary resonances. Between these two resonances, a long period—1* T* interwell solution branch is observed. This Branch F belongs to Zone A in Fig. 11. It means that a disconnected bandwidth exists, and stable solutions of Branch F are separated into two ranges. Within the disconnection, Branch H, period—1* T* asymmetrical interwell motion, borders on the left stable region and the symmetry-breaking bifurcation point of Branch F as shown in “Isolated branch of symmetrical interwell motion” section. Figure 18 shows that Branch F has limitations for bridging because the branch reaches none of the resonant regions and performance bandwidth is interrupted by the disconnection.

**Figure 19:**
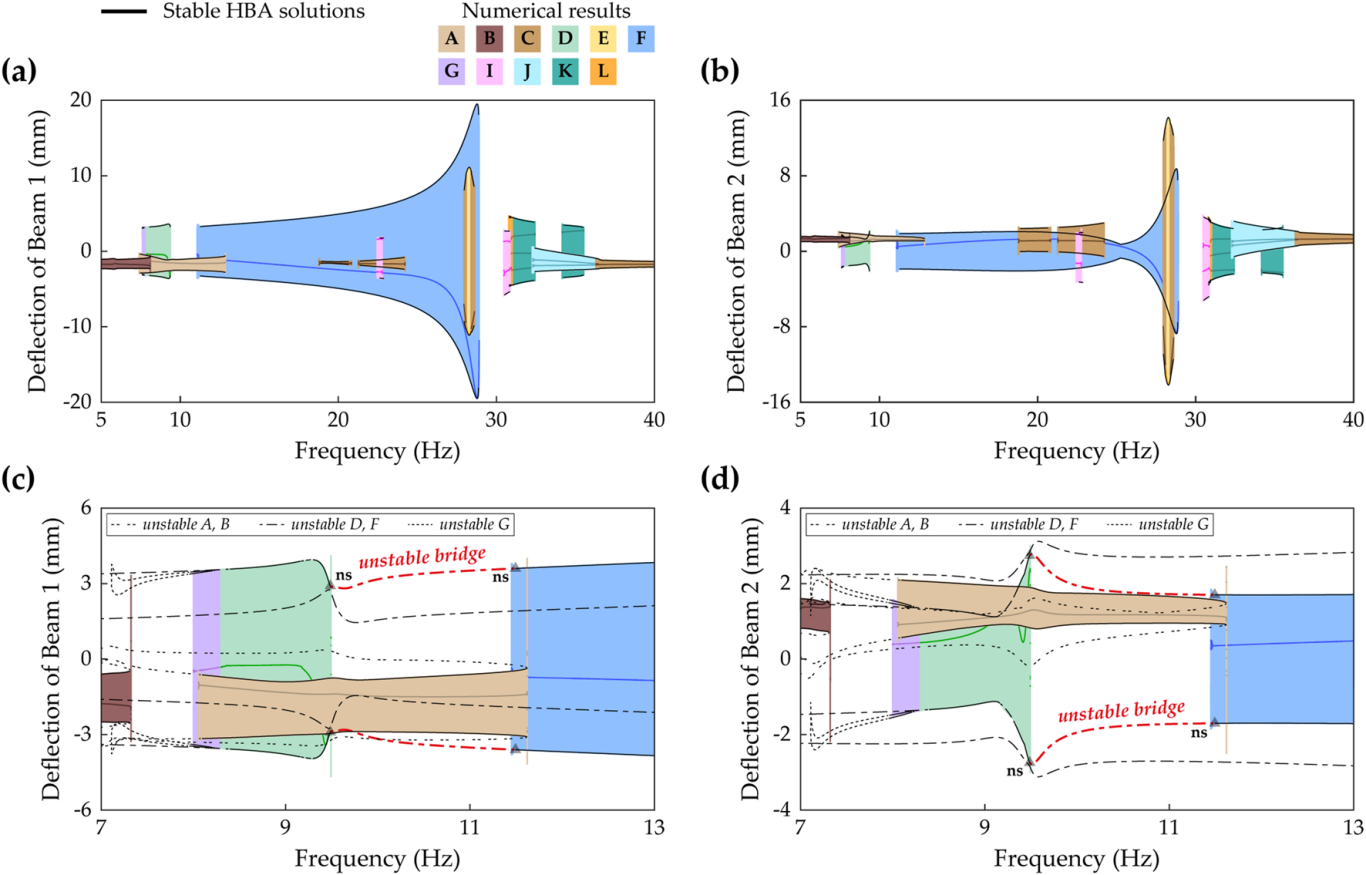
Frequency responses of all periodic solution branches in Case D (Table 1). Refer to Table 2 for branch categorization. The first row **(a,b)** and the second row **(c,d)** are the results when the base accelerations are 11 m/s^2^ and 16 m/s^2^, respectively. **(a,b)** Show sweep responses, stroboscopic points, and HBA solutions of Beam 1 and Beam 2, respectively. Unstable HBA solutions are suppressed in the figure for simplicity. In **(c,d)**, the graphs are enlarged to show that Branch D and Branch F are connected under the stronger base excitation. The solid line represents stable HBA solutions, and other lines indicate unstable HBA solutions of each branch as follows: double-dotted line—Branch A and Branch B, dot-dashed line—Branch D and Branch F, dotted line—Branch G. The triangle markers indicate the bifurcation points as follow: *ns* Neimark–Sacker bifurcation. The denotation, ‘unstable bridge’, in red color is discussed in the text.

Figure 19a,b show frequency responses investigated by the proposed HBA in Case D (beam length ratio = 0.914) when the base acceleration is 11 m/s^2^. The first and second primary resonances are found at lower frequencies than the resonances in Case C previously shown in Fig. 18. In the first primary resonance, the period—1* T* intrawell oscillations are obtained along Branch A and Branch B. The branch architectures of interwell oscillations in Branch D and Branch G have the same characteristics as the architectures in Fig. 7. In the second resonance, Branch C coalesces into Branch E, and the narrow stable range of Branch E is identified. Although Branch I is distributed over the resonant region, only two small regions are stable. It is noteworthy that the stable solutions of the period—3* T* interwell oscillations in Branch K or Branch L are obtained on the right side of the second primary resonance. It implies that period—3* T* solution branches are expected not to help bridging behavior. In contrast, Branch F belongs to Zone B in Fig. 11 and spreads over the wide frequency range including the second primary resonance. Nevertheless, Branch F is slightly short on the left side to reach a first primary resonant branch. Figure 19c,d show enlarged pictures of the branches when the base acceleration is increased to 16 m/s^2^ in order to reveal bridging dynamics between Branch D and Bridge F. Under this strong excitation, the left unstable branch tail of Branch F is connected to the turning point of Branch D at which the bifurcation was the saddle-node bifurcation. After the connection is accomplished, the bifurcation in Branch D becomes a Neimark–Sacker bifurcation. With the above-mentioned changes, it is identified that the bridge branch between Branch D and Branch F is unstable, as denoted by ‘unstable bridge’ in Fig. 19c,d. Due to these instabilities, when forward and backward sweeps are performed from Branch D and Branch F, respectively, discontinuous jumps onto Branch A after short ranges of quasi-periodic oscillations are confirmed. This unstable bridge is a flaw in bridging behavior accomplished by Branch F in Zone B in which the left unstable branch persists after the Neimark–Sacker bifurcation.

**Figure 20:**
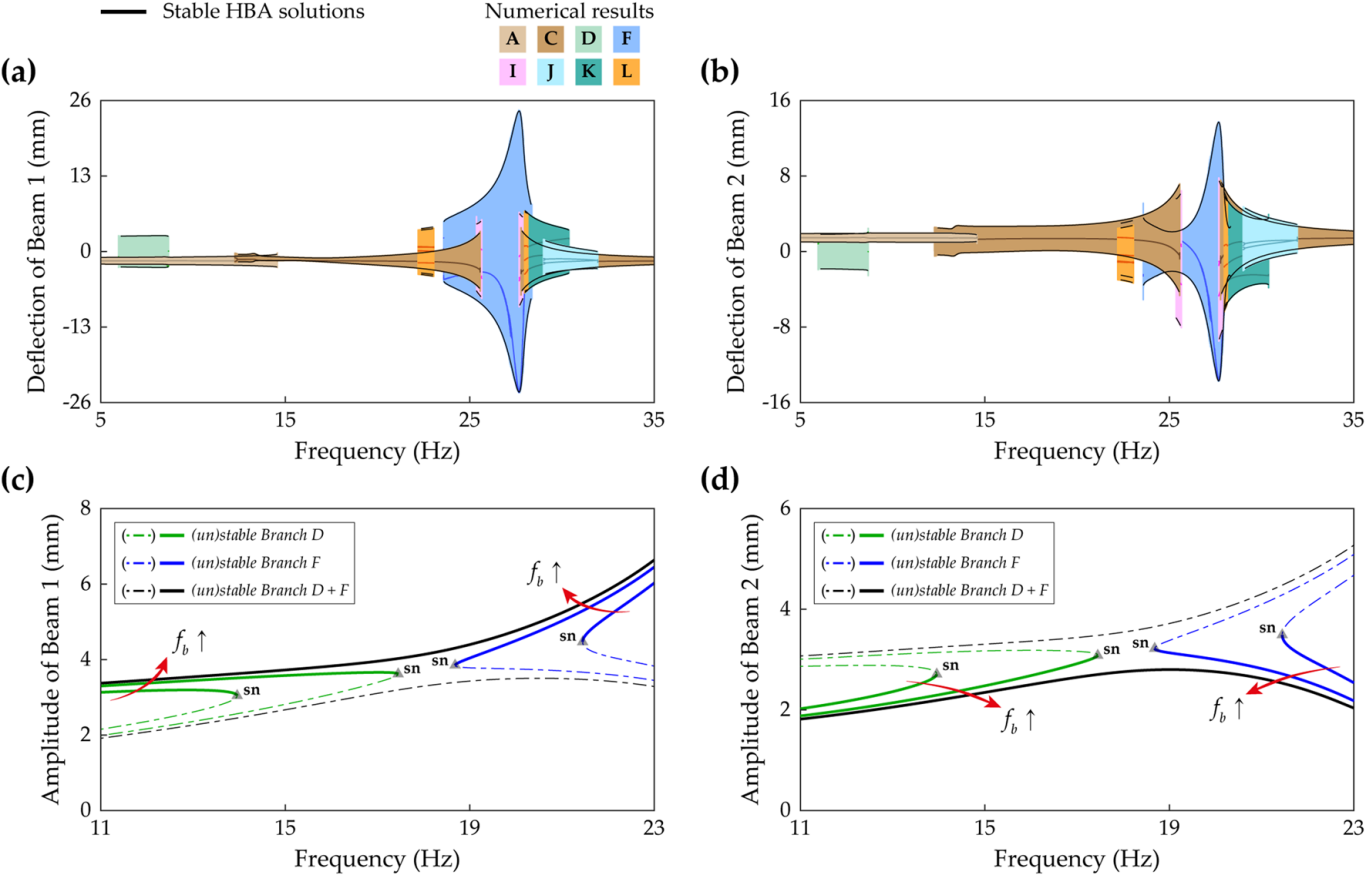
**(a,b)** Frequency responses of all periodic solution branches in Case E (Table 1) when the base acceleration is 12 m/s^2^. Refer to Table 2 for branch categorization. **(a,b)** Show sweep responses, stroboscopic points, and HBA solutions of Beam 1 and Beam 2, respectively. Unstable HBA solutions are suppressed in the figure for simplicity. **(c,d)** HBA results of Branch D and Branch F as the base acceleration increases. The base accelerations are 16, 18, and 19 m/s^2^. **(c,d)** Show Branch D and Branch F are connected when the base acceleration is 19 m/s^2^. Stable and unstable HBA solutions are plotted by the solid line and dot-dashed line, respectively. The triangle markers indicate the bifurcation points as follow: *sn* saddle-node bifurcation.

Figure 20a,b show frequency responses of all periodic oscillation branches in Case E (beam length ratio = 0.971) solved by the proposed HBA when the base acceleration is 12 m/s^2^. In the first primary resonance, a route, from Branch A to Branch D with a discontinuous jump, is not observed. In this regard, because the interwell motion is overlooked in the first primary resonance if frequency sweep algorithm is employed along the intrawell motion in Case E, an HBA is required to reveal the interwell dynamics. In the second primary resonance, Branch E, interwell motion branch, is separated from Branch C as demonstrated previously in Fig. 16, and the stability analysis confirms that Branch E is unstable across the entire frequency range. Instead, the only conspicuous branch is Branch F which belongs to Zone C in Fig. 11. Although Branch I, period—2* T* asymmetrical interwell oscillation, coexists, the stable ranges are narrow when compared to the stable range of Branch F. The period—3* T* motion is found at both sides of the second primary resonance with complicated hysteresis. As investigated in “Isolated branch of symmetrical interwell motion” section, Branch F in Zone C is placed locally in the second primary resonance. Due to this distribution of Branch F, broadband performance in Case E looks not promising when compared to Fig. 19a,b. In fact, when the base excitation becomes stronger, Branch D is prolonged towards the right and connected to Branch F. Figure 20c,d demonstrate this bridging behavior by Branch D and Branch F under 16, 18, and 19 m/s^2^ of the base accelerations. Branch D has a saddle-node bifurcation at the right turning point, and likewise, Branch F in Zone C has a saddle-node bifurcation at the left turning point. As the base acceleration increases, these two bifurcation points become closer, and eventually, Branch D and Branch F are connected. In Fig. 19c,d, when Branch F in Zone B was connected, the instabilities were caused on the bridge. Therefore, when it comes to the bridging behavior under strong excitation, the branch configuration in Zone C is superior to the one in Zone B.

## Discussion

This paper is focused on reporting the nonlinear behaviors, which are essential to understand the complicated dynamics of the 2-DOF MCBEHs, by performing the HBA. The ansatz employed in the proposed HBA involved the high-order harmonics and multiple periods in order to resolve the limitations of the single-harmonic solution. This paper provided two concrete examples in which the single-harmonic solution led to the wrong conclusion that decrement in RMS voltage output of Beam 2 for stronger excitation limits energy harvesting performance. In fact, for these examples, it has been demonstrated that when the HBA included the high-order harmonics, the decrement was incorrect or negligible. Especially for Beam 2 in the first primary resonance, the harmonic distortions produced significant effects on the response, but the HBA based on the single-harmonic solution neglected these high-order effects, thereby leading to the wrong results. Another limitation of the single-harmonic solution was the incapacity to describe asymmetrical interwell motion with respect to **x** = **0** and multiple periodic motions. As demonstrated in this paper, the structures of the asymmetrical oscillation branches and the multiple periodic oscillation branches should be identified to understand the dynamical behavior, reveal the coexisting solutions, and disclose the complete architecture of the frequency response function.

This paper has reported the asymmetrical interwell solution branch in the first primary resonance, Branch G. This branch was important to understand the bifurcation structure of the first primary resonance. When the symmetrical interwell motion, Branch D, became unstable due to a symmetry-breaking bifurcation, Branch G should be identified in order to fully comprehend the first primary resonant behavior. In the second primary resonance, the period—2* T* asymmetrical interwell solution branch, Branch I, has been reported. Because Branch I coexisted with other solution branches in isolated high-energy orbit, the HBA was suitable to reveal the dynamics of Branch I. If the frequency sweep technique is employed along the intrawell oscillation, Branch I is not captured. Moreover, by applying the HBA, the branch architecture of Branch C—intrawell oscillation and Branch E—interwell oscillation was revealed. When Branch C coalesced into Branch E at the saddle-node bifurcation and the symmetry-breaking bifurcation, stable motion of Branch E was found between these two bifurcation points. When Branch E was separated from Branch C, the stability analysis confirmed the instabilities of Branch E.

In addition, Branch F, the symmetrical interwell oscillation distributed across the long frequency range, has been reported. The configurations of Branch F were categorized into four zones according to the beam length ratio. When Branch F belonged to Zone A, the stable ranges were separated with the disconnection frequency bandwidth. In Zone B, the continuously stable architecture was obtained without the disconnection, and this branch is promising for energy harvesting for its wideband stable bandwidth. In Zone C or Zone D, Branch F was placed locally in the second primary resonance. The important difference between Zone C and Zone D was the left bifurcation structure. Whereas Branch F in Zone C had the saddle-node bifurcation at the left turning point, Branch F in Zone D had the Neimark–Sacker bifurcation on the left side and the unstable solution which persisted to the left turning point. Furthermore, bridging behavior between Branch D and Branch F was studied because period—3* T* oscillation branches were inappropriate for bridging behavior. When Branch F belonged to Zone B, Branch F had the left unstable branch tail after the Neimark–Sacker bifurcation until the left turning point. When this unstable range reached the saddle-node bifurcation point of Branch D, the saddle-node bifurcation was changed to the Neimark–Sacker bifurcation, and two branches were connected. However, the unstable bridge was developed between two Neimark–Sacker bifurcation points. In contrast, Branch F, which belonged to Zone C with the left saddle-node bifurcation, built the stable bridge when the saddle-node bifurcation points of Branch D and Branch F met.

For the 2-DOF MCBEHs, the HBA is a promising strategy to discover coexisting solutions, reveal dynamical architecture in frequency response space, disclose underlying physics of bridging behavior, and implement parameter study with fast computation. For the validity of HBA-informed results, direct numerical integration results should be compared. In the future, the findings discovered in this paper will be possibly referred to in the design process of the 2-DOF MCBEHs. Because the 2-DOF MCBEH has the complicated dynamics with the high-order harmonics, solution truncation should be conducted carefully when an HBA is performed. In the meantime, it is noted that, because the HBA is semi-analytical analysis with the Newton–Raphson method to solve algebraic equations, fully analytical interpretations on the nonlinear phenomena remain elusive. In addition, the HBA is not able to satisfy scientific curiosity about aperiodic oscillations because a solution form describes periodic motion with the Fourier series.

## Supplementary Information

Supplementary Information.
