# Supplementary material for: Harmonic balance analysis of magnetically coupled two-degree-of-freedom bistable energy harvesters
Source: Sci Rep. 2022 Apr 13;12:6221. doi: 10.1038/s41598-022-10061-x (PMC9008063; doi:10.1038/s41598-022-10061-x)
Supplement: Supplementary file 1 — Supplementary Information. [file 41598_2022_10061_MOESM1_ESM.pdf]

Supplementary Information

Harmonic Balance Analysis of Magnetically Coupled Two-degree-of-freedom Bistable Energy Harvesters

Jinhong Noh, Minh Sang Nguyen, Pilkee Kim, and Yong-Jin Yoon

J.N. (jinhongnoh@kaist.ac.kr)
M.S.N. (minhsang.08clc@gmail.com)
P.K. (pkim@jbnu.ac.kr)
Y.-J.Y. (yongjiny@kaist.ac.kr)

This section explains how the approximate steady-state periodic solution is obtained using the harmonic balance method. The oscillator model equations, Eq. (1) in Section 2, are written out first in the following form:

$$0 = \ddot{w}_1 + 2\zeta_1\omega_1\dot{w}_1 + \omega_1^2 w_1 - \beta_1 V_1 - k_{11}w_1 - k_{12}w_2 - k_{13}w_1^3 - k_{14}w_1^2 w_2 - k_{15}w_1 w_2^2 - k_{16}w_2^3 + \alpha_1 f_b \cos(\Omega t)\,, \tag{S1-a}$$

$$0 = \ddot{w}_2 + 2\zeta_2\omega_2\dot{w}_2 + \omega_2^2 w_2 - \beta_2 V_2 - k_{21}w_1 - k_{22}w_2 - k_{23}w_1^3 - k_{24}w_1^2 w_2 - k_{25}w_1 w_2^2 - k_{26}w_2^3 + \alpha_2 f_b \cos(\Omega t)\,, \tag{S1-b}$$

$$0 = \dot{V}_1 + \eta_1 V_1 + \gamma_1 \dot{w}_1\,, \tag{S1-c}$$

$$0 = \dot{V}_2 + \eta_2 V_2 + \gamma_2 \dot{w}_2\,. \tag{S1-d}$$

The main purpose of solving process is to make all residuals, the right-hand sides above, zeros. Herein, k_{ij} terms were given from the Taylor series expansion of the magnetic coupling force, which is position-dependent functions. Considering that the nonlinearities are polynomials and the excitation is periodic with frequency Ω, one may seek a solution form as

$$w_1(t) \approx a_{10} + a_{1c}\cos(\Omega t) + a_{1s}\sin(\Omega t)\,, \tag{S2-a}$$

$$w_2(t) \approx a_{20} + a_{2c}\cos(\Omega t) + a_{2s}\sin(\Omega t)\,, \tag{S2-b}$$

$$V_1(t) \approx b_{10} + b_{1c}\cos(\Omega t) + b_{1s}\sin(\Omega t)\,, \tag{S2-c}$$

$$V_2(t) \approx b_{20} + b_{2c}\cos(\Omega t) + b_{2s}\sin(\Omega t)\,. \tag{S2-d}$$

This assumed solution form is substituted into Eq. (S1) and expanded. Subsequently, the following trigonometric identities are utilized to reduce a high-power trigonometric term to a sum of trigonometric functions:

$$\sin^2 \Omega t = (1 - \cos 2\Omega t)/2\,, \tag{S3-a}$$

$$\cos^2 \Omega t = (1 + \cos 2\Omega t)/2\,, \tag{S3-b}$$

$$\cos^3 \Omega t = (3\cos \Omega t + \cos 3\Omega t)/4\,, \tag{S3-c}$$

$$\sin^3 \Omega t = (3\sin \Omega t - \sin 3\Omega t)/4\,. \tag{S3-d}$$

Because Eq. (S2) has truncated up to the first harmonic, the balancing process for each harmonic term is conducted with neglecting high-order terms. The first mechanical part of the model, Eq (S1-a), yields the following relations from the constant term, cosine term, and sine term balances, respectively:

$$\begin{aligned} 0 = {} & \omega_1^{\,2} a_{10} - \beta_1 b_{10} - k_{11}a_{10} - k_{12}a_{20} \\ & - k_{13}a_{10}^{\;3} - \frac{3k_{13}}{2}a_{10}\left(a_{1c}^{\;2} + a_{1s}^{\;2}\right) - k_{14}a_{10}^{\;2}a_{20} - k_{14}a_{10}\left(a_{1c}a_{2c} + a_{1s}a_{2s}\right) - \frac{k_{14}}{2}a_{20}\left(a_{1c}^{\;2} + a_{1s}^{\;2}\right) \\ & - k_{16}a_{20}^{\;3} - \frac{3k_{16}}{2}a_{20}\left(a_{2c}^{\;2} + a_{2s}^2\right) - k_{15}a_{10}a_{20}^{\;2} - k_{15}a_{20}\left(a_{1c}a_{2c} + a_{1s}a_{2s}\right) - \frac{k_{15}}{2}a_{10}\left(a_{2c}^{\;2} + a_{2s}^2\right), \end{aligned} \tag{S4-a}$$

$$\begin{aligned} 0 = {} & -\Omega^2 a_{1c} + 2\Omega\zeta_1\omega_1 a_{1s} - \beta_1 b_{1c} + \omega_1^{\,2} a_{1c} + \alpha_1 f_b - k_{11}a_{1c} - k_{12}a_{2c} \\ & - 3k_{13}a_{10}^{\;2}a_{1c} - \frac{3k_{13}}{4}a_{1c}\left(a_{1c}^{\;2} + a_{1s}^{\;2}\right) - k_{14}a_{10}\left(a_{10}a_{2c} + 2a_{20}a_{1c}\right) - \frac{k_{14}}{2}a_{1c}a_{1s}a_{2s} - \frac{k_{14}}{4}\left(3a_{1c}^{\;2} + a_{1s}^{\;2}\right)a_{2c} \\ & - 3k_{16}a_{20}^{\;2}a_{2c} - \frac{3k_{16}}{4}a_{2c}\left(a_{2c}^{\;2} + a_{2s}^2\right) - k_{15}a_{20}\left(a_{20}a_{1c} + 2a_{10}a_{2c}\right) - \frac{k_{15}}{2}a_{1s}a_{2c}a_{2s} - \frac{k_{15}}{4}a_{1c}\left(3a_{2c}^{\;2} + a_{2s}^{\;2}\right), \end{aligned} \tag{S4-b}$$

$$\begin{aligned} 0 = {} & -\Omega^2 a_{1s} - 2\Omega\zeta_1\omega_1 a_{1c} - \beta_1 b_{1s} + \omega_1^{\,2} a_{1s} - k_{11}a_{1s} - k_{12}a_{2s} \\ & - 3k_{13}a_{10}^{\;2}a_{1s} - \frac{3k_{13}}{4}a_{1s}\left(a_{1c}^{\;2} + a_{1s}^{\;2}\right) - k_{14}a_{10}\left(a_{10}a_{2s} + 2a_{20}a_{1s}\right) - \frac{k_{14}}{2}a_{1c}a_{1s}a_{2c} - \frac{k_{14}}{4}\left(a_{1c}^{\;2} + 3a_{1s}^2\right)a_{2s} \\ & - 3k_{16}a_{20}^{\;2}a_{2s} - \frac{3k_{16}}{4}a_{2s}\left(a_{2c}^{\;2} + a_{2s}^{\;2}\right) - k_{15}a_{20}\left(a_{20}a_{1s} + 2a_{10}a_{2s}\right) - \frac{k_{15}}{2}a_{1c}a_{2c}a_{2s} - \frac{k_{15}}{4}a_{1s}\left(a_{2c}^2 + 3a_{2s}^2\right). \end{aligned} \tag{S4-c}$$

Likewise, the second mechanical part provides

$$0=\omega_2{}^2a_{20}-\beta_2b_{20}-k_{21}a_{10}-k_{22}a_{20}$$
$$-k_{23}a_{10}{}^3-\frac{3k_{23}}{2}a_{10}\left(a_{1c}{}^2+a_{1s}{}^2\right)-k_{24}a_{10}{}^2a_{20}-k_{24}a_{10}\left(a_{1c}a_{2c}+a_{1s}a_{2s}\right)-\frac{k_{24}}{2}a_{20}\left(a_{1c}{}^2+a_{1s}{}^2\right) \quad \text{(S5-a)}$$
$$-k_{26}a_{20}{}^3-\frac{3k_{26}}{2}a_{20}\left(a_{2c}{}^2+a_{2s}{}^2\right)-k_{25}a_{10}a_{20}{}^2-k_{25}a_{20}\left(a_{1c}a_{2c}+a_{1s}a_{2s}\right)-\frac{k_{25}}{2}a_{10}\left(a_{2c}{}^2+a_{2s}{}^2\right),$$
$$0=-\Omega^2a_{2c}+2\Omega\zeta_2\omega_2a_{2s}-\beta_2b_{2c}+\omega_2{}^2a_{2c}+\alpha_2f_b-k_{21}a_{1c}-k_{22}a_{2c}$$
$$-3k_{23}a_{10}{}^2a_{1c}-\frac{3k_{23}}{4}a_{1c}\left(a_{1c}{}^2+a_{1s}{}^2\right)-k_{24}a_{10}\left(a_{10}a_{2c}+2a_{20}a_{1c}\right)-\frac{k_{24}}{2}a_{1c}a_{1s}a_{2s}-\frac{k_{24}}{4}\left(3a_{1c}^2+a_{1s}{}^2\right)a_{2c} \quad \text{(S5-b)}$$
$$-3k_{26}a_{20}{}^2a_{2c}-\frac{3k_{26}}{4}a_{2c}\left(a_{2c}{}^2+a_{2s}{}^2\right)-k_{25}a_{20}\left(a_{20}a_{1c}+2a_{10}a_{2c}\right)-\frac{k_{25}}{2}a_{1s}a_{2c}a_{2s}-\frac{k_{25}}{4}a_{1c}\left(3a_{2c}{}^2+a_{2s}^2\right),$$
$$0=-\Omega^2a_{2s}-2\Omega\zeta_2\omega_2a_{2c}-\beta_2b_{2s}+\omega_2{}^2a_{2s}-k_{21}a_{1s}-k_{22}a_{2s}$$
$$-3k_{23}a_{10}{}^2a_{1s}-\frac{3k_{23}}{4}a_{1s}\left(a_{1c}{}^2+a_{1s}{}^2\right)-k_{24}a_{10}\left(a_{10}a_{2s}+2a_{20}a_{1s}\right)-\frac{k_{24}}{2}a_{1c}a_{1s}a_{2c}-\frac{k_{24}}{4}\left(a_{1c}{}^2+3a_{1s}{}^2\right)a_{2s} \quad \text{(S5-c)}$$
$$-3k_{26}a_{20}{}^2a_{2s}-\frac{3k_{26}}{4}a_{2s}\left(a_{2c}{}^2+a_{2s}{}^2\right)-k_{25}a_{20}\left(a_{20}a_{1s}+2a_{10}a_{2s}\right)-\frac{k_{25}}{2}a_{1c}a_{2c}a_{2s}-\frac{k_{25}}{4}a_{1s}\left(a_{2c}{}^2+3a_{2s}{}^2\right).$$

The two electric parts also give Eq. (S6) and Eq. (S7), respectively:

$$0=\eta_1b_{10}, \quad \text{(S6-a)}$$
$$0=\Omega b_{1s}+\eta_1b_{1c}+\Omega\gamma_1a_{1s}, \quad \text{(S6-b)}$$
$$0=-\Omega b_{1c}+\eta_1b_{1s}-\Omega\gamma_1a_{1c}, \quad \text{(S6-c)}$$

and

$$0=\eta_2b_{20}, \quad \text{(S7-a)}$$
$$0=\Omega b_{2s}+\eta_2b_{2c}+\Omega\gamma_2a_{2s}, \quad \text{(S7-b)}$$
$$0=-\Omega b_{2c}+\eta_2b_{2s}-\Omega\gamma_2a_{2c}. \quad \text{(S7-c)}$$

Assuming Eq. (S2), therefore, leads to 12 algebraic equations (4×3 = 12) for 12 unknowns (*a* and *b* in Eq. (S2)).

To account for high-order harmonics, an ansatz is readily extended from Eq. (S2) to

$$w_1(t)\approx a_{10}+\sum_{k=1}^{H}a_{1ck}\cos\left(k\Omega t\right)+a_{1sk}\sin\left(k\Omega t\right), \quad \text{(S8-a)}$$
$$w_2(t)\approx a_{20}+\sum_{k=1}^{H}a_{2ck}\cos\left(k\Omega t\right)+a_{2sk}\sin\left(k\Omega t\right), \quad \text{(S8-b)}$$
$$V_1(t)\approx b_{10}+\sum_{k=1}^{H}b_{1ck}\cos\left(k\Omega t\right)+b_{1sk}\sin\left(k\Omega t\right), \quad \text{(S8-c)}$$
$$V_2(t)\approx b_{20}+\sum_{k=1}^{H}b_{2c}\cos\left(k\Omega t\right)+b_{2s}\sin\left(k\Omega t\right), \quad \text{(S8-d)}$$

where *H* denotes the truncation order. Subsequently, one can repeat the walk-through of the balancing process above paragraph. In this case, the number of unknowns is given as 4×(1+2*H*); e.g., 44 unknowns for *H* = 5. Thus, four nonlinear ordinary differential equations reduce to 44 nonlinear algebraic equations for the fifth-order truncation. Until now, the response has been assumed to be *T*-periodic. In general, however, a nonlinear system can show multiple-periodic motion. Because the period *T* equals 2π/Ω, one may seek period—*nT* solution in the following form:

$$w_1(t)\approx a_{10}+\sum_{k=1}^{H}a_{1ck}\cos\left(\frac{k\Omega t}{n}\right)+a_{1sk}\sin\left(\frac{k\Omega t}{n}\right), \quad \text{(S9-a)}$$

$$w_2(t) \approx a_{20} + \sum_{k=1}^{H} a_{2ck} \cos\left(\frac{k\Omega t}{n}\right) + a_{2sk} \sin\left(\frac{k\Omega t}{n}\right), \tag{S9-b}$$

$$V_1(t) \approx b_{10} + \sum_{k=1}^{H} b_{1ck} \cos\left(\frac{k\Omega t}{n}\right) + b_{1sk} \sin\left(\frac{k\Omega t}{n}\right), \tag{S9-c}$$

$$V_2(t) \approx b_{20} + \sum_{k=1}^{H} b_{2c} \cos\left(\frac{k\Omega t}{n}\right) + b_{2s} \sin\left(\frac{k\Omega t}{n}\right), \tag{S9-d}$$

where n is a positive integer. For example, in order to obtain period—3T response, n is set to be 3. In the same manner, this assumption also leads to 4×(1+2H) nonlinear algebraic equations. This study mainly employs H = 5. It follows that the number of equations to solve is 44. Finally, these algebraic equations can be solved using the Newton-Raphson algorithm with appropriate initial conditions. ■
